# Rare complement factor I variants associated with reduced macular thickness and age-related macular degeneration in the UK Biobank

**DOI:** 10.1093/hmg/ddac060

**Published:** 2022-03-14

**Authors:** Nikolaos Tzoumas, David Kavanagh, Heather J Cordell, Andrew J Lotery, Praveen J Patel, David H Steel

**Affiliations:** Biosciences Institute, Newcastle University, Newcastle upon Tyne, United Kingdom; Complement Therapeutics Research Group, Translational and Clinical Research Institute, Newcastle University, Newcastle upon Tyne, United Kingdom; Complement Therapeutics Research Group, Translational and Clinical Research Institute, Newcastle University, Newcastle upon Tyne, United Kingdom; National Renal Complement Therapeutics Centre, Royal Victoria Infirmary, Newcastle upon Tyne, United Kingdom; Population Health Sciences Institute, Newcastle University, Newcastle upon Tyne, United Kingdom; Clinical & Experimental Sciences, Faculty of Medicine, University of Southampton, Southampton, United Kingdom; NIHR Biomedical Research Centre at Moorfields Eye Hospital NHS Foundation Trust and UCL Institute of Ophthalmology, London, United Kingdom; Biosciences Institute, Newcastle University, Newcastle upon Tyne, United Kingdom; Sunderland Eye Infirmary, Sunderland, United Kingdom

## Abstract

To evaluate potential diagnostic and therapeutic biomarkers for age-related macular degeneration (AMD), we identified 8433 UK Biobank participants with rare complement Factor I gene (*CFI*) variants, 579 with optical coherence tomography-derived macular thickness data. We stratified these variants by predicted gene expression and measured their association with retinal pigment epithelium-Bruch’s membrane (RPE-BM) complex and retinal thicknesses at nine macular subfields, as well as AMD risk, using multivariable regression models adjusted for the common complement Factor H gene (*CFH*) p.Y402H and age-related maculopathy susceptibility protein 2 gene (*ARMS2*) p.A69S risk genotypes. *CFI* variants associated with low Factor I levels predicted a thinner mean RPE-BM (95% confidence interval [CI] −1.66 to −0.37 μm, *P* = 0.002) and retina (95% CI −5.88 to −0.13 μm, *P* = 0.04) and a higher AMD risk (odds ratio [OR] = 2.26, 95% CI 1.56 to 3.27, *P* < 0.001). *CFI* variants associated with normal Factor I levels did not impact mean RPE-BM/retinal thickness (*P* = 0.28; *P* = 0.99) or AMD risk (*P* = 0.97). *CFH* p.Y402H was associated with a thinner RPE-BM (95% CI −0.31 to −0.18 μm, *P* < 0.001 heterozygous; 95% CI −0.62 to −0.42 μm, *P* < 0.001 homozygous) and retina (95% CI −0.73 to −0.12 μm, *P* = 0.007 heterozygous; 95% CI −1.08 to −0.21 μm, *P* = 0.004 homozygous). *ARMS2* p.A69S did not influence RPE-BM (*P* = 0.80 heterozygous; *P* = 0.12 homozygous) or retinal thickness (*P* = 0.75 heterozygous; *P* = 0.07 homozygous). p.Y402H and p.A69S exhibited a significant allele–dose response with AMD risk. Thus, *CFI* rare variants associated with low Factor I levels are robust predictors of reduced macular thickness and AMD. The observed association between macular thickness and *CFH* p.Y402H, but not *ARMS2* p.A69S, highlights the importance of complement dysregulation in early pathogenesis.

## Introduction

Rare variants are increasingly recognized as an important source of genetic variation underpinning age-related macular degeneration (AMD), the commonest cause of irreversible sight loss in the developed world (1). Prior studies indicate a strong relationship between AMD and mutations in the complement Factor I gene (*CFI*) that encodes Factor I, the key negative regulatory enzyme of complement (1–7). Complement overactivation is hypothesized to result in subretinal para-inflammation which drives AMD progression in predisposed individuals (8,9), and is thought to account for the majority of the genetic susceptibility to the condition (10). However, the sequence of biological changes that define the transition between normal ageing and early disease are unclear (11). Establishing predictor variables of AMD in the context of complement dysregulation is crucial in selecting patients most likely to benefit from complement therapeutics, several of which are currently progressing through late-stage clinical development (8).

Factor I is a serum serine protease that cleaves serum and cell-bound C3b and C4b in the presence of cofactors [e.g. Factor H, complement component 4 binding protein (C4BP), monocyte chemoattractant protein-1 (MCP) and complement receptor type 1 (CR1)] (9). In doing so, Factor I inhibit all pathways of complement, particularly the intrinsically active alternative pathway that is thought to initiate AMD (12,13). There is systemic and local synthesis of Factor I, with most produced by the liver (9). Retinal pigment epithelium (RPE) cells, Müller cells and retinal neurons are considered the main sources of complement transcripts in the posterior pole of the eye (14,15). As there is a large gradient in Factor I levels across the blood-aqueous barrier (3), and it has been demonstrated that Bruch’s membrane (BM) is impermeable to Factor I (16), it is likely that most Factor I in the eye is locally produced. Three types of *CFI* rare variants are described: Type 1 variants reduce serum Factor I levels by around 50% even in the heterozygous state and are strongly associated with AMD (3,5,6). Type 2 variants result in normal Factor I levels but impaired regulatory function; their role in AMD is less clear (5,17). Both type 1 and type 2 *CFI* variants may lead to unregulated complement activation and consumptive loss of complement component 3 (C3) predisposing to non-resolving inflammation in various tissues (18). A third group consists of variants of uncertain significance (VUS) that are associated with normal serum levels but have an unknown impact on Factor I function (17). As Factor I mediates the effects of other endogenous complement inhibitors implicated in AMD, notably Factor H, establishing the ophthalmic manifestations of *CFI* type 1 variants can yield important diagnostic and therapeutic biomarkers for complement gene defects in general (5). However, the rarity of these genetic variants has so far precluded a systematic assessment of their effects on retinal microstructure.

Optical coherence tomography (OCT) is a rapid, non-invasive imaging modality which provides cross-sectional structural information about the neurosensory retina and RPE to an axial resolution of 3–8 μm (19). OCT-derived metrics of macular thickness are associated with AMD risk factors such as ageing and smoking (20,21), and are under increased scrutiny as proxies for systemic inflammation and microvascular dysfunction (19). The UK Biobank is the largest biorepository of retinal imaging to-date, encompassing over 130 000 OCT scans, many of which are segmented for RPE–BM complex and/or retinal thickness at the macula. This provides a unique opportunity to characterize the macular phenotype of patients with *CFI* rare variants (RVs) and determine the genetic interactions that influence AMD risk.

The aim of this study was to assess the effect of *CFI* RVs on the macular microstructure and AMD risk of healthy subjects. We hypothesized that carriers of *CFI* type 1 RVs would be predisposed to thinner macular RPE–BM and neural retinal layers and be at a higher risk of AMD compared with carriers of *CFI* VUS. We were also interested to ascertain the interaction between any additional risks conferred by *CFI* type 1 RVs and other well-known risk factors for AMD, including the common genetic variants complement Factor H gene (*CFH*) p.Y402H and age-related maculopathy susceptibility protein 2 gene (*ARMS2*) p.A69S. Finally, we sought to clarify the association between *CFI* RVs and membranoproliferative glomerulonephritis (MPGN) and thrombotic microangiopathy (TMA) which encompass, respectively, the pathological entities of C3 glomerulopathy (C3G) and atypical hemolytic uremic syndrome (aHUS) that are exemplars of systemic complement deposition (22).

## Results

### Basic demographics

**Figure 1:**
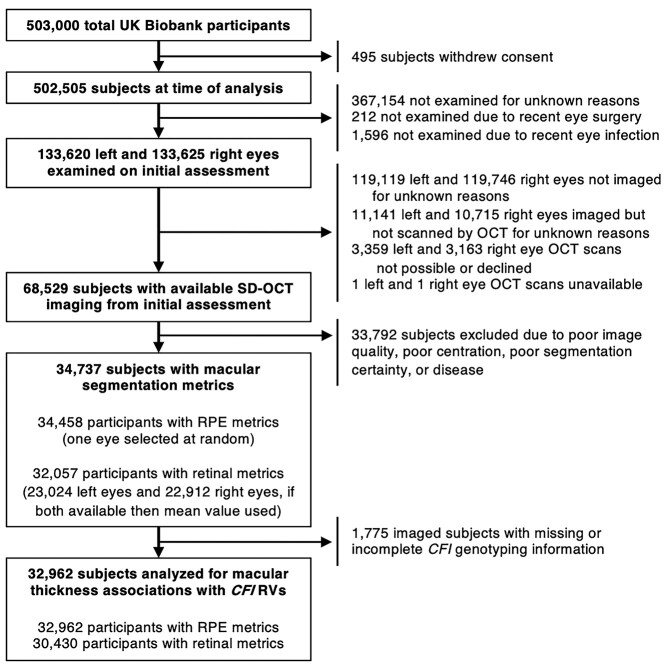
Flowchart showing exclusion criteria for RPE-BM and retinal macular thickness association analyses involving *CFI* RVs. Abbreviations: *CFI* = complement factor I gene, RPE-BM = retinal pigment epithelium-Bruch’s membrane complex, RVs = rare variants, SD-OCT = spectral domain optical coherence tomography.

A total of 502 505 UK Biobank participants’ data were available at the time of analysis. Of these, 133 543 subjects underwent eye examination and 68 529 had spectral domain-OCT (SD-OCT) macular imaging. After the exclusion of 33 792 participants who did not meet the criteria for automatic segmentation of OCT images and 1775 (5.11%) participants with missing *CFI* genotype data, the sample size for analysis of RPE–BM or retinal macular thickness in the context of *CFI* RVs was 32 962 (Fig. 1). Of these, 87 (0.26%) subjects carried a *CFI* type 1 variant, 8 (0.02%) carried p.I340T (the only *CFI* type 2 variant represented in the UK Biobank) and 493 (1.49%) carried a *CFI* VUS. Seventy participants were homozygous for a *CFI* RV, only two of which had OCT-derived macular thickness measures (Table 1). Twenty-seven participants were compound heterozygotes for two different variants in the *CFI* gene, but it was not possible to establish whether these were present on the same or different alleles (Supplementary Material, Table S1). No participant was heterozygous for three or more of the selected *CFI* variants. The baseline characteristics of participants in *CFI* type 1 and VUS groups are shown in Table 2. The prevalence of *CFI* type 1 variants did not change significantly with increasing age in either the overall or imaged populations (Supplementary Material, Fig. S2).

**Table 1:** Distribution of individuals with *CFI* rare variants in the UK Biobank

*CFI* variant		Number of participants			
Class	Variant	Overall (*n* = 502 505)		With macular RPE-BM or retinal thickness metrics (*n* = 34 737)	
		Heterozygous	Homozygous	Heterozygous	Homozygous
Type 1 (*n* = 1671)	p.G119R	962	1	58	0
	p.G287R	333	0	14	0
	p.A240G	97	0	4	0
	p.I357M	92	0	2	0
	p.H418L	66	0	3	0
	p.G162D	45	0	1	0
	p.A431T	29	0	0	0
	p.R474X	24	0	3	0
	p.P50A	9	0	2	0
	p.G188V	4	0	0	0
Type 2	p.I340T	128	0	8	0
VUS (*n* = 6788)	p.G261D	2659	1	192	0
	p.R406H	2521	63	193	2
	p.K441R	1536	5	106	0
	p.R345Q	3	0	0	0

Abbreviations: *CFI* = complement factor I gene, OCT = optical coherence tomography, VUS = variant of uncertain significance.

**Table 2:** Baseline characteristics of participants with OCT-derived macular thickness metrics. *P*-values for hypothesis testing were computed using an F-test for continuous data and Fisher’s exact test for categorical data

Variable	*CFI* type 1 RV carriers (*n* = 87)	*CFI* VUS carriers (*n* = 492)	*P-*value
Mean age at recruitment, years (SD)	55.8 (8.4)	54.5 (8.5)	0.20
Mean age at death, years (SD)	63.4 (4.1)	67.0 (5.3)	0.34
Female gender (%)	40 (46.5)	256 (52.4)	0.35
Ethnicity (%)			<0.001
White	82 (96.5)	344 (71.2)	
Mixed	3 (3.5)	8 (1.7)	
Asian	0 (0.0)	69 (14.3)	
Black	0 (0.0)	4 (0.8)	
Chinese	0 (0.0)	28 (5.8)	
Other	0 (0.0)	30 (6.2)	
Smoking status (%)			0.56
Never	50 (58.8)	307 (62.8)	
Previous	28 (32.9)	133 (27.2)	
Current	7 (8.2)	49 (10.0)	
Mean Townsend index (SD)	−1.1 (2.9)	−0.9 (2.8)	0.68
Mean BMI, kg/m^2^ (SD)	27.2 (4.5)	26.5 (4.2)	0.18
Mean VA, logMAR (SD)	−0.0 (0.1)	−0.0 (0.1)	0.44
Mean IOPg, mmHg (SD)	15.3 (3.2)	14.9 (3.0)	0.34
Mean refraction, D (SD)	−0.5 (2.0)	−0.4 (1.9)	0.49
Mean systolic BP, mmHg (SD)	135.2 (15.1)	134.1 (18.6)	0.63
Mean SCr, μmol/L (SD)	74.7 (15.1)	71.9 (15.1)	0.14
Mean CRP, mg/L (SD)	2.0 (2.2)	2.2 (3.6)	0.55
Mean HbA1c, mmol/mol (SD)	34.7 (3.8)	35.6 (4.2)	0.08
*CFH* p.Y402H (%)			0.06
*Heterozygous*	44 (51.2)	214 (43.9)	
*Homozygous*	16 (18.6)	64 (13.1)	
*ARMS2* p.A69S (%)			0.98
*Heterozygous*	34 (39.5)	186 (38.0)	
*Homozygous*	6 (7.0)	37 (7.6)	

*Abbreviations*: BMI = body mass index, BP = blood pressure, *CFI* = complement Factor I gene, CRP = C-reactive protein, D = diopters, HbA1c = glycated hemoglobin, IOPg = Goldmann-corrected intraocular pressure, logMAR = logarithm of the minimum angle of resolution, OCT = optical coherence tomography, RV = rare variant, SCr = serum creatinine, SD = standard deviation, VA = visual acuity.

### Macular thickness associations

The mean macular RPE–BM and retinal thicknesses among all included participants were 25.3 μm (SD 2.9 μm) and 278.5 μm (SD 13.0 μm), respectively. Mean RPE–BM and retinal thicknesses by age and genotype are shown in Tables 3 and 4. Univariable linear regression analysis indicated that increased mean RPE–BM thickness is significantly associated with decreased mean retinal thickness at the macula (*B* = −0.11 μm, 95% confidence interval [CI] −0.16 to −0.06; *P* < 0.001). Using multivariable stepwise linear regression, we observed a significant association between increasing age and a thinner mean RPE–BM (unstandardized coefficient [*B*] = −0.06 μm for each year, 95% CI −0.06 to −0.05; *P* < 0.001) and retina (*B* = −0.28 μm for each year, 95% CI −0.30 to −0.26; *P* < 0.001) at the macula. We also observed a significant association between current smoking status and a thinner mean retina (*B* = −0.90 μm, 95% CI −1.39 to −0.41; *P* < 0.001) but not RPE–BM (*P* = 0.60).

**Table 3:** Macular RPE-BM thickness of imaged participants in the UK Biobank by age and genotype. Comparisons are highlighted with respect to WT genotype using Welch Two Sample *t*-tests without adjustments. Significant differences are indicated by ^*^for *P* < 0.05, ^**^for *P* < 0.01 and ^***^for *P* < 0.001

Age (years)	Mean RPE-BM thickness, μm (SD)								
	*CFI* variants			*CFH* p.Y402H			*ARMS2* p.A69S		
	WT (*n* = 32 110)	Type 1 (*n* = 85)	VUS (*n* = 486)	WT (*n* = 12 878)	Heterozygous (*n* = 15 438)	Homozygous (*n* = 4757)	WT (*n* = 20 095)	Heterozygous (*n* = 11 409)	Homozygous (*n* = 1627)
<50	25.9 (3.2)	25.2 (2.6)	25.8 (3.3)	26.0 (3.3)	25.9 (3.2)^*^	25.7 (3.1)^**^	25.9 (3.2)	26.0 (3.3)	26.1 (3.1)
50 to 59	25.3 (2.8)	24.5 (2.2)	24.8 (2.4)^*^	25.5 (2.9)	25.2 (2.8)^***^	24.9 (2.7)^***^	25.3 (2.8)	25.3 (2.8)	25.0 (2.7)^*^
≥60	24.9 (2.5)	23.4 (1.6)^***^	24.8 (2.6)	25.1 (2.6)	24.8 (2.5)^***^	24.6 (2.4)^***^	25.0 (2.5)	24.9 (2.5)	24.8 (2.4)

Abbreviations: *ARMS2* = age-related maculopathy susceptibility protein 2 gene, *CFH* = complement Factor H gene, *CFI* = complement Factor I gene, SD = standard deviation, VUS = variant of uncertain significance.

**Table 4:** Macular retinal thickness of imaged participants in the UK Biobank by age and genotype. Comparisons are highlighted with respect to WT genotype using Welch Two Sample *t*-tests without adjustments. Significant differences are indicated by ^*^for *P* < 0.05, ^**^for *P* < 0.01 and ^***^for *P* < 0.001

Age (years)	Mean retinal thickness, μm (SD)								
	*CFI* variants			*CFH* p.Y402H			*ARMS2* p.A69S		
	**WT (*n* = 29 891)**	**Type 1 (*n* = 77)**	**VUS (*n* = 452)**	**WT (*n* = 11 906)**	**Heterozygous (*n* = 14 420)**	**Homozygous (*n* = 4451)**	**WT (*n* = 18 747)**	**Heterozygous (*n* = 10 579)**	**Homozygous (*n* = 1506)**
<50	280.0 (12.9)	277.9 (13.1)	278.6 (12.2)	280.0 (12.7)	280.1 (13.2)	280.4 (12.9)	280.0 (12.9)	280.0 (13.2)	279.4 (11.7)
50 to 59	279.0 (12.9)	278.5 (14.9)	278.8 (14.7)	279.0 (12.8)	279.2 (13.1)	278.6 (12.9)^*^	279.0 (12.9)	279.0 (13.0)	278.6 (13.6)
≥60	277.0 (12.9)	270.7 (12.0)^*^	276.2 (13.5)	277.0 (12.7)	276.4 (13.0)^*^	276.4 (12.9)	277.0 (13.0)	276.7 (12.7)	275.0 (13.3)^**^

Abbreviations: *ARMS2* = age-related maculopathy susceptibility protein 2 gene, *CFH* = complement factor H gene, *CFI* = complement Factor I gene, SD = standard deviation, VUS = variant of uncertain significance.

**Figure 2:**
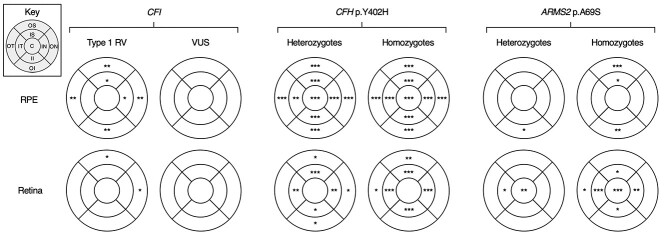
Significance of reduced thickness in OCT-derived metrics across the macula. Significance of multivariable testing for different genotypes and RPE-BM (above) or retinal (below) thinning in each ETDRS macular subfield using stepwise linear regression. Significant differences are indicated by ^*^for *P* < 0.05, ^**^for *P* < 0.01 and ^***^for *P* < 0.001. Abbreviations: *ARMS2* = age-related maculopathy susceptibility protein 2 gene, C = central macular subfield*, CFH* = complement factor H gene, *CFI* = complement Factor I gene, II = inner inferior subfield, IN = inner nasal subfield, IS = inner superior subfield, IT = inner temporal subfield, OI = outer inferior subfield, ON = outer nasal subfield, OS = outer superior subfield, OT = outer temporal subfield, RV = rare variant, VUS = variant of uncertain significance.

Using stepwise linear regression, we found a significant association between *CFI* type 1 RV carrier status and a thinner mean RPE–BM (*B* = −1.01 μm, 95% CI −1.66 to −0.37 μm; *P* = 0.002) as well as at the inner superior, inner nasal and all outer Early Treatment Diabetic Retinopathy Study (ETDRS) macular subfields (Fig. 2). *CFI* type 1 RV carrier status was also associated with a thinner mean retina (*B* = −3.01 μm, 95% CI −5.88 to −0.13 μm; *P* = 0.04) as well as at the outer superior and outer nasal subfields (Fig. 2). *CFI* VUS carrier status was not significantly associated with a change in mean RPE–BM or retinal thickness at the macula (*B* = −0.15 μm, 95% CI −0.41 to 0.12 μm; *P* = 0.28 for RPE–BM, and *B* = 0.01 μm, 95% CI −1.18 to 1.20 μm; *P* = 0.99 for retina) or at any subfield (Fig. 2). The distribution of RPE–BM and retinal thicknesses at each macular subfield for *CFI* type 1 RV and VUS carriers is presented in Supplementary Material, Figure S3. Regression coefficients for individual and grouped variants at each macular subfield are also available in the Supplementary Material, Appendix 1.

*CFH* p.Y402H genotype was associated with a thinner mean macular RPE–BM overall (*B* = −0.25 μm, 95% CI −0.31 to −0.18 μm; *P* < 0.001 for heterozygotes, and *B* = −0.52 μm, 95% CI −0.62 to −0.42 μm; *P* < 0.001 for homozygotes) and across all subfields compared with wild-type (WT; Fig. 2). p.Y402H was also associated with a thinner retina on average (*B* = −0.42 μm, 95% CI −0.73 to −0.12 μm; *P* = 0.007 for heterozygotes, and *B* = −0.64 μm, 95% CI −1.08 to −0.21 μm; *P* = 0.004 for homozygotes) and across all inner subfields at the macula (Fig. 2). Additionally, p.Y402H heterozygosity was associated with thinner outer superior, nasal and inferior subfields of the retina, and p.Y402H homozygosity was associated with thinner outer temporal and superior subfields of the retina at the macula (Fig. 2).

*ARMS2* p.A69S was not significantly associated with a difference in mean RPE–BM (*B* = −0.01 μm, 95% CI −0.08 to 0.06 μm; *P* = 0.80 for heterozygotes, and *B* = −0.12 μm, 95% CI −0.27 to 0.03 μm; *P* = 0.12 for homozygotes) or retinal (*B* = 0.05 μm, 95% CI, −0.25 to 0.35 μm; *P* = 0.75 for heterozygotes, and *B* = −0.62 μm, 95% CI −1.28 to 0.05 μm; *P* = 0.07 for homozygotes) macular thickness compared with WT. However, p.A69S heterozygosity was significantly associated with a thinner RPE–BM at the outer inferior subfield and with a thinner retina in the central and inner temporal subfields of the macula (Fig. 2). Additionally, p.A69S homozygosity was significantly associated with a thinner RPE–BM at the inner and outer superior subfields, and the outer inferior position (Fig. 2). Finally, p.A69S homozygosity was significantly associated with a thinner retina at the central and outer temporal positions, and all inner subfields of the macula (Fig. 2).

### Exploring the relationship between mean macular thickness, age and genotype

Combined RPE–BM and retinal thicknesses at the macula were thinner with increasing age across all genotype groups (−1.5 μm between <50 and 50–59-year-olds, versus −3.0 μm between the 50–59 and ≥60-year-olds; Tables 3 and 4). The greatest difference in mean RPE–BM thickness across age groups was between <50 and 50–59-year-olds for all at-risk genotypes except *CFI* type 1 RV, in whom the observed change between <50 and 50–59-year-olds (−0.7 μm) was less than that between 50–59 and ≥60-year-olds (−1.1 μm). In contrast, the greatest difference in mean retinal thickness across age groups was observed between 50–59 and ≥60-year-olds across all genotypes, especially in *CFI* type 1 RV (−3.7 μm) and *ARMS2* p.A69S homozygous (−3.8 μm) groups.

**Figure 3:**
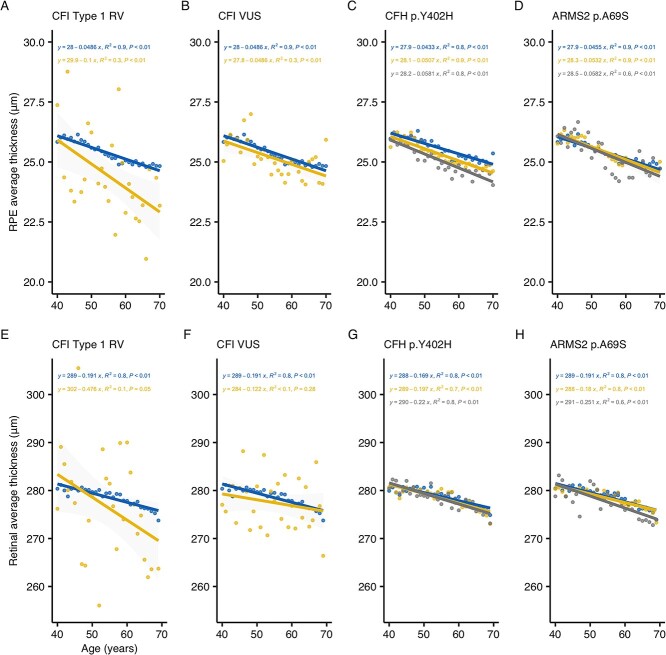
Relationship of mean macular RPE and retinal thickness with age. Scatter plots of mean RPE-BM (**A**–**D**) and retinal (**E**–**H**) thickness versus age for *CFI* type 1 RV (A, E), *CFI* VUS (B, F), *CFH* p.Y402H (C, G) and *ARMS2/HTRA1* risk haplotype (D, H). Genotype groups are indicated by color (blue for non-carriers/WT, yellow for *CFI* RV carriers and p.Y402H or p.A69S heterozygotes, and dark gray for p.Y402H or p.A69S homozygotes). Each plotted point represents the mean RPE-BM or retinal macular thickness for each genotype group at each year of age. Linear model regression lines and 95% CIs (light gray bands) are also shown. Fitted coefficients, *R*^2^ and *P*-values are indicated above each plot. Abbreviations: *ARMS2* = age-related maculopathy susceptibility protein 2 gene, *CFH* = complement Factor H gene, *CFI* = complement factor I gene, RV = rare variant, VUS = variant of uncertain significance.

To further explore the relationship between age, genotype, and macular thickness, we plotted mean RPE–BM/retinal thicknesses at each year of age, stratified by genotype, and fitted linear regression models to these (Fig. 3). Next, we used simple slopes analyses to evaluate whether genotype influenced the association between age and mean RPE–BM/retinal thickness (Supplementary Material, Table S2). There was an appreciable but non-significant difference between the association of mean RPE–BM (*P =* 0.38) and retinal (*P =* 0.13) thickness with age between *CFI* type 1 RV carriers and non-carriers (Fig. 3). There was no noticeable difference between the association of mean RPE–BM (*P =* 0.68) and retinal (*P =* 0.34) thickness with age between *CFI* VUS carriers and non-carriers (Fig. 3). There was also no significant difference in the association of mean RPE–BM or retinal macular thickness and age between *CFI* type 1 RV and VUS carriers (*P =* 0.50 for RPE–BM and *P =* 0.08 for retina, Supplementary Material, Fig. S4). Additionally, we found no significant difference in the association of mean RPE–BM or retinal macular thickness and age between WT, heterozygous and homozygous states of *CFH* p.Y402H (Supplementary Material, Table S2), although we observed early evidence of an allele–dose divergence in the gradients of our regression lines for mean RPE–BM thickness (Fig. 3). Similarly, we found no significant difference in the association of mean RPE–BM or retinal macular thickness and age between WT and homozygous, or heterozygous and homozygous, states of *ARMS2* p.A69S (Supplementary Material, Table S2), but observed an allele–dose divergence in gradient for mean RPE–BM thickness (Fig. 3). Finally, we found a significant difference in the association of mean RPE–BM thickness and age between *ARMS2* p.A69S WT and heterozygous participants (*P* = 0.03).

### Outcome analyses

Of the total UK Biobank population, 3950 participants (0.78%, 95% CI 0.76 to 0.81) had received an inpatient diagnosis of AMD at the time of recruitment, which is less than the prevalence of advanced AMD in similar populations (23). The prevalence of AMD among all *CFI* type 1 RV carriers (1.7%) was greater than that among *CFI* VUS carriers (0.8%). The proportion of *CFI* type 1 RV carriers among AMD cases was 0.73%, compared with 0.33% among non-AMD cases. In contrast, the proportion of *CFI* VUS carriers was the same for both AMD and non-AMD cases (1.3%). No *CFI* RV compound heterozygotes were diagnosed with AMD (Supplementary Material, Table S1).

The mean age of participants diagnosed with AMD was 63.1 years (SD = 5.7) for *CFI* type 1 RV carriers, 62.2 (4.8) for *CFI* VUS carriers, 62.9 (5.6) and 63.4 (5.0) for *CFH* p.Y402H heterozygotes and homozygotes, respectively, 63.0 (5.5) and 63.8 (5.0) for *ARMS2* p.A69S heterozygotes and homozygotes, respectively, compared with 63.0 (5.5) across the entire population. An overview of AMD prevalence by age and genotype is presented in Table 5.

**Table 5:** Prevalence of AMD in the UK Biobank by age and genotype. Comparisons are highlighted with respect to WT genotype using Chi-squared tests without adjustments. Significant differences are indicated by ^*^for *P* < 0.05, ^**^for *P* < 0.01 and ^***^for *P* < 0.001

Age (years)	AMD prevalence, % (95% CI)								
	*CFI* variants			*CFH* p.Y402H			*ARMS2* p.A69S		
	WT	Type 1	VUS	WT	Heterozygous	Homozygous	WT	Heterozygous	Homozygous
<50	0.1 (0.1–0.1)	0.5 (0–1.2)	0.1 (0–0.2)	0.1 (0.1–0.1)	0.1 (0.1–0.2)	0.1 (0.0–0.1)	0.1 (0.1–0.1)	0.1 (0.1–0.2)	0.1 (0.0–0.2)
50 to 59	0.4 (0.4–0.4)	0.5 (0–1.1)	0.5 (0.2–0.8)	0.3 (0.3–0.4)	0.4 (0.4–0.5)^*^	0.5 (0.4–0.6)^**^	0.4 (0.3–0.4)	0.4 (0.4–0.5)	0.7 (0.5–0.9)^***^
≥60	1.4 (1.4–1.5)	3.4 (2.1–4.8)^***^	1.5 (1.0–1.9)	1.3 (1.2–1.3)	1.4 (1.3–1.5)^*^	2.1 (1.9–2.3)^***^	1.3 (1.2–1.3)	1.6 (1.5–1.7)^***^	3.0 (2.6–3.3)^***^

Abbreviations: *ARMS2* = age-related maculopathy susceptibility protein 2 gene, *CFH* = complement Factor H gene, *CFI* = complement Factor I gene, VUS = variant of uncertain significance.

**Figure 4:**
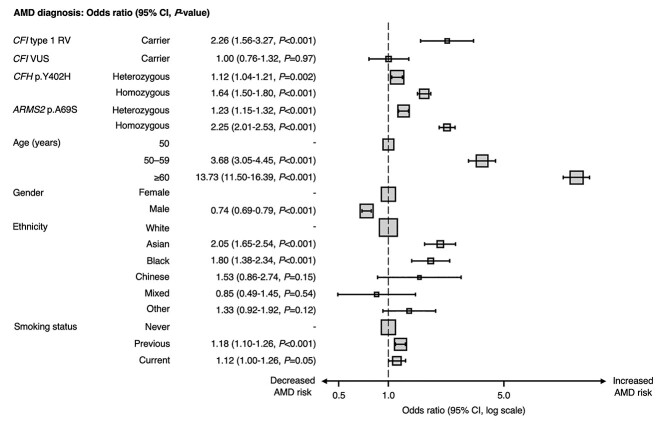
Forest plot of odds ratios for AMD diagnosis from multilevel (mixed effects) logistic regression analysis adjusted for age, gender, ethnicity, smoking status and genotype as fixed effects, and recruitment site as a random effect. Abbreviations: *ARMS2* = age-related maculopathy susceptibility protein 2 gene, *CFH* = complement Factor H gene, *CFI* = complement Factor I gene, RV = rare variant, VUS = variant of uncertain significance.

Multilevel logistic regression confirmed that *CFI* type 1 RV carrier status was significantly associated with a higher odds ratio (OR) of AMD (OR 2.26, 95% CI 1.56 to 3.27; *P* < 0.001). We also showed that harboring a *CFI* VUS did not influence the odds of AMD in either direction (OR 1.00, 95% CI 0.76 to 1.32; *P* = 0.97). Additionally, we confirmed an allele–dose response of *CFH* p.Y402H and *ARMS2* p.A69S on AMD risk (Fig. 4). Univariable and multivariable ORs resulting from this analysis are available in Supplementary Material, Table S3.

Among the 24 participants with a health record-derived diagnosis of TMA and 47 with MPGN, no *CFI* type 1 or type 2 RV carriers were represented. One *CFI* VUS carrier (p.G261D heterozygous) had a diagnosis of MPGN type 1 or 3, but none had a diagnosis of TMA.

### Gene interaction analyses

Finally, we entered the product terms of *CFH* p.Y402H, *ARMS2* p.A69S and *CFI* type 1 RV or VUS genotypes into our multivariable logistic regression model (with age, gender, ethnicity, smoking status and genotype as independent variables and AMD diagnosis as the dependent variable) to explore whether the effect of each risk genotype on AMD risk would differ in the presence of other genotypes (Table 6). This revealed a significant interaction between *CFI* type 1 RV carrier status and *ARMS2* p.A69S homozygosity (OR 7.03, 95% CI 0.90 to 40.61; *P* = 0.04), indicating that the combined effect of these genotypes is seven times the product of the individual effects of each genotype separately. In contrast, there was no significant interaction between *CFI* type 1 RV carrier status and *CFH* p.Y402H homozygosity (OR 0.58, 95% CI 0.03 to 4.02; *P* = 0.63). There were also no significant interaction effects between *CFI* type 1 RV and *ARMS2* p.A69S heterozygous (OR 1.29, 95% CI 0.25 to 5.98; *P* = 0.74) or *CFH* p.Y402H heterozygous (OR 1.45, 95% CI 0.45 to 5.49; *P* = 0.55) states on AMD risk. In addition, we found highly significant interactions between *CFH* p.Y402H and *ARMS2* p.A69S on AMD risk when either genotype existed in the homozygous state (Table 6).

**Table 6:** Influence of genotype interactions on AMD risk. Odds ratios of multivariable logistic regression analyses for AMD are adjusted for age, gender, ethnicity, smoking status and *CFI* type 1 RV, *CFI* VUS, *CFH* and *ARMS2* genotypes as fixed effects. Products of genotype terms were entered into our model to check for interactions between genotypes. Only the coefficients for two-way interactive terms are shown. Significant differences are indicated by ^*^for *P* < 0.05, ^**^for *P* < 0.01 and ^***^for *P* < 0.001

Genotype interaction		*N*	OR multivariable (95% CI, *P-*value)
*Genotype 1*	*Genotype 2*		
*CFI* Type 1 RV	*CFH* p.Y402H heterozygous^†^	811	1.45 (0.45–5.49, *P* = 0.55)
*CFI* Type 1 RV	*CFH* p.Y402H homozygous^†^	230	0.58 (0.03–4.02, *P* = 0.63)
*CFI* Type 1 RV	*ARMS2* p.A69S heterozygous^†^	563	1.29 (0.25–5.98, *P* = 0.74)
*CFI* Type 1 RV	*ARMS2* p.A69S homozygous^†^	68	7.03 (0.90–40.61, *P* = 0.04)^*^
*CFI* VUS	*CFH* p.Y402H heterozygous^‡^	2934	0.46 (0.17–1.12, *P* = 0.10)
*CFI* VUS	*CFH* p.Y402H homozygous^‡^	838	0.65 (0.18–1.84, *P* = 0.45)
*CFI* VUS	*ARMS2* p.A69S heterozygous^‡^	2468	0.81 (0.33–1.88, *P* = 0.63)
*CFI* VUS	*ARMS2* p.A69S homozygous^‡^	438	1.10 (0.61–1.96, *P* = 0.75)
*CFH* p.Y402H heterozygous	*ARMS2* p.A69S heterozygous^†^	78 082	1.14 (0.97–1.33, *P* = 0.11)
*CFH* p.Y402H heterozygous	*ARMS2* p.A69S homozygous^†^	11 036	1.73 (1.31–2.30, *P* < 0.001)^***^
*CFH* p.Y402H homozygous	*ARMS2* p.A69S heterozygous^†^	24 098	1.35 (1.11–1.64, *P* = 0.003)^**^
*CFH* p.Y402H homozygous	*ARMS2* p.A69S homozygous^†^	3359	2.23 (1.60–3.10, *P* < 0.001)^***^

^†^Not adjusting for *CFI* VUS.

^‡^Not adjusting for *CFI* type 1 RV.

Abbreviations: *ARMS2* = age-related maculopathy susceptibility protein 2 gene, *CFH* = complement Factor H gene, *CFI* = complement Factor I gene, OR = odds ratio, VUS = variant of uncertain significance.

### Sensitivity analyses

To test whether our results are influenced by population structure and interindividual relatedness, we undertook a genetically informed sensitivity analysis using two complementary approaches: (1) a linear mixed model score test as implemented in the RVTESTS package (24), which uses an empirical kinship matrix to account for close or distant ancestry between individuals; and (2) a two-step approach implemented within the regenie package (25), which uses a whole-genome ridge regression approach in Step 1 to generate an adjusted test of association at Step 2. As an additional sensitivity check, we also repeated these analyses removing all participants that had been flagged by UK Biobank as being of ‘non-white’ ethnicity. This reduced the number of individuals contributing to the analysis to 23 994 (for mean RPE-BM thickness) or 24 206 (for mean retinal thickness) for RVTESTS, or 26 185 for regenie, reflecting differences in the handling of missing covariate data between packages as detailed in our Materials and Methods.

Results from the multivariable analyses with RVTESTS and regenie confirmed the associations seen between *CFI* type 1 RVs and mean RPE–BM (*P* = 0.002 with RVTESTS, *P* = 0.002 with regenie) and mean retinal thickness (*P* = 0.025 with RVTESTS, *P* = 0.015 with regenie), as well as the lack of association seen between *CFI* VUS and mean RPE–BM (*P* = 0.61 with RVTESTS, *P* = 0.62 with regenie) or mean retinal thickness (*P* = 0.76 with RVTESTS, *P* = 0.44 with regenie). Removal of non-white individuals from the analysis retained the significance of the associations seen between *CFI* type 1 RVs and mean RPE–BM (*P* = 0.001 with RVTESTS, *P* = 0.002 with regenie) but reduced the significance of the associations seen between *CFI* type 1 RVs and mean retinal thickness (*P* = 0.071 with RVTESTS, *P* = 0.11 with regenie), while retaining the lack of association between *CFI* VUS and mean RPE–BM (*P* = 0.10 with RVTESTS, *P* = 0.25 with regenie) or mean retinal thickness (*P* = 0.44 with RVTESTS, *P* = 0.54 with regenie).

To test whether these findings were independent of rs10033900, a common *CFI* SNP which has been associated with AMD in previous genome-wide association studies (GWAS) (1), we first established the low association between rs10033900 and our selected *CFI* type 1 RVs (*R*^2^ = 0.0003; *D*′ = 0.45) and VUS (*R*^2^ = 0.0001, *D*′ = 0.14). To further confirm that this common marker does not influence our findings, we repeated our analyses in RVTESTS to include the rs10033900 genotype (coded as a two-level factor) and found that our associations were essentially unchanged for both *CFI* type 1 RVs (*P* = 0.002 for mean RPE-BM thickness, *P* = 0.022 for mean retinal thickness) and VUS (*P* = 0.63 for mean RPE-BM thickness, *P* = 0.76 for mean retinal thickness).

Testing for association between *CFI* rare variants (type 1 or VUS) and the binary AMD disease phenotype in the full UK Biobank cohort using RVTESTS or regenie proved too computationally prohibitive for our computer system, so we used a simpler approach whereby we removed all non-white individuals and one of each pair of related (with kinship >3rd degree) individuals. This left 321 389 individuals for analysis using multivariable logistic regression (glm function in R) with age, gender, smoking status and genotypes at *CFH* p.Y402H and *ARMS2* p.A69S (both coded as two-level factors) as covariates. Results confirmed the associations seen between *CFI* type 1 RVs and AMD (OR 2.30, 95% CI 1.47 to 3.59, *P* < 0.001) as well as the lack of association seen between *CFI* VUS and AMD (OR 0.78, 95% CI 0.50 to 1.23, *P* = 0.29). Models including interaction terms in this smaller dataset found no significant interactions between *CFI* type 1 RVs or VUS and *CFH* (*P* = 0.39 between *CFI* type 1 RVs and *CFH* p.Y402H homozygotes) or *ARMS2* (*P* = 0.47 between *CFI* type 1 RVs and p.A69S homozygotes) on AMD risk, although interactions between *CFH* p.Y402H or *ARMS2* p.A69S risk genotypes were significant (*P* = 0.021 for *CFH* p.Y402H homozygotes and *ARMS2* p.A69S heterozygotes, *P* = 0.036 for *CFH* p.Y402H heterozygotes and *ARMS2* p.A69S homozygotes, and *P* < 0.001 for double homozygosity).

## Discussion

In our multisite community-based study, we examined the impact of risk alleles in the *CFI*, *CFH* and *ARMS2/HTRA1* (HtrA serine peptidase 1 gene) genes on RPE–BM and retinal thicknesses at the macula, and analyzed their interaction on AMD risk. We demonstrated strong associations between thinner RPE–BM/retinal layers and the principal AMD risk factors of increasing age and smoking (20,21), and identified novel associations of genetic complement defects with macular thickness (Fig. 2). Mean macular RPE–BM and retinal thicknesses are comparable at 40 years of age but may diverge progressively thereafter in high-risk genotypes (Fig. 3), for example, reaching significance in *CFI* type 1 RV carriers over 60 years of age (Tables 3 and 4). Therefore, the reduced thickness of these layers in at-risk participants is likely to reflect accelerated ageing processes rather than developmental differences. In fact, we showed that mean RPE–BM thinning is most apparent between <50 and 50–59-year-olds while mean retinal thinning is greatest between 50–59 and ≥60-year-olds (Tables 3 and 4). These observations are in line with our appreciation of the RPE–BM as a primary site of AMD pathology and imply that its degeneration or dysfunction precedes loss of overlying photoreceptors (Fig. 3) (26).

In line with previous work, we showed that *CFI* type 1 RVs increase AMD risk independently of demographic and common genetic factors (Fig. 4) (1,3,5–7). We also showed that these variants result in the thinnest RPE–BM/retina at all ages relative to other genotypes (Tables 3 and 4), and that this process may begin as early as 40 years (Fig. 3). Using multivariable linear regression, we demonstrated that these variants predict reduced macular thickness independently of age and other demographic or genetic factors (Fig. 2). Compared with the common *CFH* p.Y402H variant, each *CFI* null allele predisposed to a 4- and 8-fold greater reduction in mean macular RPE–BM and retinal thicknesses, respectively. Indeed, the reduction in mean RPE–BM and retinal thickness associated with one *CFI* type 1 RV is equivalent to that of 17 and 11 years of ageing, respectively, in healthy participants. Furthermore, we showed that RPE–BM/retinal atrophy associated with these RVs is most apparent in the macular periphery, especially the superior and nasal subfields (Fig. 2). This topography is similar to the reported distribution of reticular pseudodrusen in nonexudative AMD (27,28), which segregate with genetic complement defects such as *CFI* p.G119R and are associated with overlying RPE–BM effacement and rod dysfunction—unfortunately, image grading data are currently unavailable to verify this in our population (29,30). Although we do not replicate the younger age of AMD onset among *CFI* type 1 RV carriers reported by Saskens and colleagues (Table 5), familial aggregation studies are by nature highly segregated for ancestry and are, therefore, confounded by unmapped genetic and environmental elements (30). This issue is minimized in the UK Biobank through the random selection of participants, and through our genetically informed sensitivity analyses. Finally, *CFI* type 1 RV prevalence does not change with age, implying that there is no link to mortality (Supplementary Material, Fig. S2).

Photoreceptor and RPE atrophy are features of both ageing and AMD (31), but these are accelerated and predict disease progression in the latter (32). *CFI*-mediated reduced macular thickness may reflect vascular rarefaction and cellular atrophy in retinal tissues in the context of complement overactivation—as described in preclinical models of retinal degeneration and post-mortem eyes with geographic atrophy (12,33–35). As RPE cell density decreases and rod density increases toward the macular periphery (36,37), both phenotypes of a thinner macula and reticular pseudodrusen may be related to the impaired upregulation of *CFI* expression by RPE cells and subsequent failure to protect rod photoreceptors from complement overactivation (9). Our findings reinforce the theory that AMD results from cumulative pro-inflammatory insults at the level of the RPE (11), and that degeneration and loss of key tissues occur before the disease progresses to its advanced stages (38). This supports early pharmacological intervention to maximize the amount of viable tissue and pre-empt detrimental chronic inflammation (11).

As hypothesized, *CFI* VUS are not associated with AMD or with a thinner mean RPE and retina at the macula (Fig. 2), suggesting that the selected variants have a negligible effect on gene function. Indeed, multiple studies have shown that *CFI* p.G261D, p.R406H, p.K441R and p.R345Q predominantly result in normal Factor I levels (3,5,7) (Supplementary Material, Table S4).

Of the 1 671 patients with *CFI* type 1 RVs, no participant has a diagnosis of aHUS—a disease previously reported to be associated with *CFI* variants. This suggests that the penetrance of *CFI* to cause aHUS is low and may rely on other genetic modifiers, consistent with the lack of large pedigrees of *CFI* RV carriers with this disease (22). There are also no *CFI* type 1 RV carriers with MPGN/C3G, in line with recent data suggesting that the condition is seldom explained by a single genetic complement defect (39). Additionally, we have not identified any significant difference in creatinine clearance between *CFI* type 1 and VUS groups (Table 2). These data highlight the importance of informed genetic stratification of patients in the design of complement therapeutic trials.

Consistent with a recent study, we found a significant association between *CFH* p.Y402H and a thinner mean RPE and retina at the macula of healthy participants (40), and further identify an additive dose effect for homozygotes (Figs 2 and 3). *CFH* p.Y402H was the first major genetic risk factor for AMD to be identified, impairing the localization of Factor H to the outer retina to protect against complement overactivation and facilitate the clearance of pro-inflammatory elements (8). In our study, *CFH* p.Y402H was significantly associated with diffuse macular thinning in a centripetal pattern similar to *CFI* type 1 RVs (Fig. 2). Although Factor H and Factor I have different tissue expression patterns, both are fluid-phase proteins that diffuse throughout the retina (8). Therefore, we may expect to see a similarly diffuse pattern of macular thinning in *CFI* type 1 RV carriers with larger cohorts.

We showed that the *ARMS2/HTRA1* risk haplotype was independently associated with AMD risk (Fig. 4), but we did not observe a significant association between *ARMS2* p.A69S and mean macular RPE/retina thickness. This contrasts the findings of Zouache *et al.* (40), who showed a significant decline in RPE/retinal thickness among homozygous *ARMS2* p.A69S subjects without AMD relative to carriers of a protective variant. However, it should be noted that our study population is two decades younger (mean age of 56 versus 74 years) and that our controls are WT participants. These study design differences are likely to have amplified the effect of *ARMS2* p.A69S observed by Zouache and colleagues. With increasing age, we may expect *ARMS2/HTRA1*-associated retinal thinness in our cohort to also extend to most ETDRS subfields, in line with our identification of a significant association between mean RPE thickness and age among heterozygous relative to WT *ARMS2/HTRA1* participants (Fig. 3). Nevertheless, the pattern of retinal thinning associated with the *ARMS2/HTRA1* risk haplotype is centrifugal and diminished compared with that of *CFI* type 1 RVs and *CFH* p.Y402H (Fig. 2, Supplementary Material, Appendix 1). This topography may reflect the effect of *ARMS2/HTRA1* on extracellular matrix remodeling (41), which may alter foveal morphology.

Using multivariable logistic regression analyses with gene interactive terms and adjusted for known predictors, we found that there is no significant interaction between *CFI* type 1 RVs and *CFH* p.Y402H on AMD risk, despite a substantial sample size (Table 6). Factor H promotes the dissociation of C3 convertase and is one of the co-factors that mediate the cleavage and inactivation of C3b by Factor I (10). In the setting of *CFI* haploinsufficiency, however, it appears that the Factor H haplotype has no effect as Factor I is limiting. This suggests that complement-associated AMD risk is principally mediated by the ability of Factor I and its co-factors to degrade C3b to inactive C3b (iC3b), rather than the effects of Factor H on C3 convertase dissociation (which is not Factor I-dependent) (10). Indeed, it has been proposed that the complement hyperinflammatory phenotype that manifests in AMD, aHUS, or C3G is dependent on iC3b generation, which is not possible in the complete absence of Factor I (10). This theory is supported by the lack of *CFI* type 1 RV homozygotes or compound heterozygotes diagnosed with these conditions in our study and in the literature (Table 1, Supplementary Material, Table S1) (22). Further interactive analyses are required to clarify the limiting enzymatic cascades in at-risk complotypes. As complement gene defects and the *ARMS2/HTRA1* risk haplotype contribute differently to AMD pathogenesis, it is expected that the presence of both would lead to a significant interaction on disease risk (Table 6), although our sensitivity analyses show that this association may not be robust to genetic confounding.

A potential criticism of the results as presented is that these analyses did not consider the known relatedness that exists between several UK Biobank participants, nor the known differences in genetic structure between subpopulations, which may result in increased type 1 error rates when testing for genotype–phenotype associations. To check sensitivity to these issues, we performed a more robust analysis of the association between *CFI* rare variants (type 1 or VUS) and the mean RPE–BM or mean retinal thickness phenotypes using the complementary analytical approaches provided by the RVTESTS and regenie software packages. This approach has been shown to perform similarly to linear mixed model approaches in terms of adjusting for ancestry. In principle, both analyses are expected to account for both close and distant levels of relatedness (i.e. family relationships and ethnicity); however, as an additional sensitivity check, we also repeated these analyses removing all participants that had been flagged by UK Biobank as being ‘non-white’. The results of these analyses with the full cohort confirmed that *CFI* type 1 RVs, but not *CFI* VUS, are associated with a reduction in mean RPE–BM and retinal thicknesses. On removing non-white individuals from our analyses, the association between *CFI* type 1 RVs and reduced mean RPE–BM thickness was robust, while the association with reduced mean retinal thickness was lost. Furthermore, after excluding all non-white individuals and one of each pair of related individuals from our cohort and repeating our multivariable logistic regression analysis, we showed that the association between *CFI* type 1 RVs and AMD is robust to genetic confounding and likely causal. However, the interaction between *CFI* type 1 RVs and *ARMS2* p.A69S status on AMD risk was less robust in this smaller cohort.

One question is whether these rare variant associations can be attributable to the known *CFI* GWAS variant rs10033900. Such an explanation seems unlikely based on the relatively low level of linkage disequilibrium seen between rs10033900 and type 1 RVs in our sample (*R*^2^ = 0.0003; *D*′ = 0.45) but, as an added check, we repeated the RVTESTS and regenie analyses including rs10033900 genotype (coded as a two-level factor) as an additional covariate in our analyses, observing negligible differences in the *P*-values obtained.

We found that the ORs for AMD due to *CFI* type 1 RV alleles in our population were lower than previously reported (1,3,5–7). As well as other *CFI* rare variants described in AMD which were not covered by the present study, there exist risk variants in *CFI* and other genes which have not yet been identified or validated in our population. As such, individuals with undetected or private rare variants will have been included in our controls, reducing the observed difference in AMD risk between groups. We have attempted to minimize this confounding by incorporating population characteristics as site of recruitment in our mixed effects model in addition to key demographic and genetic covariates, showing that the 95% CIs for these may range markedly higher in the co-presence of *CFH* p.Y402H or the *ARMS2/HTRA1* risk haplotype (Table 6). This approach resulted in favorable model fitting criteria (Supplementary Material, Table S5), notwithstanding statistical methods for the reduction of estimation bias in imbalanced datasets (Supplementary Material, Table S6). Another consideration is that the incidence of AMD in the UK Biobank cohort is lower than the sampling population, which suggests that our calculated ORs closely approximate the corresponding relative risk ratios.

Some further limitations of our study should be noted. As UK Biobank participants with self-reported AMD were excluded from automated segmentation of OCT-derived thickness metrics, we could not accurately establish the interaction between RPE or retinal macular thickness and AMD risk. We also do not have access to the segmentation data of individual tissues within the neural retina, so were unable to draw inferences on the effect of complement overactivation on individual retinal cell populations. Additionally, choroidal thickness has not been characterized in this cohort.

In this largest population analysis of *CFI* RVs to-date, we report novel genotype–phenotype associations relating to macular RPE and retinal thicknesses and risk of AMD in otherwise healthy participants that seem robust to genetic confounding. We found that reduced RPE or retinal macular thickness is a consistent marker of ageing that is magnified by *CFI* RVs associated with low serum Factor I levels, more so than the common *CFH* p.Y402H and *ARMS2* p.A69S risk genotypes. We also show that the influence of *CFI* on RPE and retinal macular thickness and disease risk is likely to be conditional on the level of functional Factor I. Moreover, our findings indicate that complement overactivation in the macula is a quantitative but extensive phenomenon that can be appreciated before the onset of AMD. Overall, these findings support the pharmacological supplementation of Factor I to prevent the progression of macular ageing processes to AMD in predisposed individuals. In future, it is possible to envisage treatments and clinical trials for AMD that are stratified by patients’ clinical and genetic characteristics. Our study informs the multifactorial pathogenesis of AMD and supports new research in this direction.

## Materials and Methods

### Demographics

The UK Biobank was a prospective community cohort study from 2006 to 2010 that collected biological data for over 500 000 people aged 40–69 years and resident in the UK during recruitment (42). The age, gender, ethnicity, smoking status and Townsend deprivation index of participants at recruitment were obtained from the UK Biobank Resource. Participants identified their ethnic background as either white, mixed, Asian or Asian British, Black or Black British, Chinese or ‘Other’, with each group accounted for separately in our principal analyses.

### Physical measures

Automated resting systolic blood pressure (SBP) readings were obtained from the UK Biobank Resource, and the mean of these was calculated for each participant. Body mass index (BMI) was also obtained from the UK Biobank Resource. Eye measures included logarithm of the minimum angle of resolution (logMAR) visual acuity, Goldmann-corrected intraocular pressure (IOPg) using the Ocular Response Analyzer® (Reichert Inc., NY) and spherical equivalent measurements from autorefraction using the RC-5000 device (Tomey, AZ) for each eye. Nonmydriatic retinal SD OCT measurements were acquired in a dim room with the Topcon 3D OCT 1000 Mk2 device (Topcon Corporation, Japan), using the 6 × 6 mm raster pattern 3D macular volume scan consisting of 128 B-scans, which each B-scan comprising 512 horizontally oriented A-scans.

### Ethical approval

The UK Biobank has approval from the North West Multi-centre Research Ethics Committee, which covers the UK, in accordance with the tenets of the Declaration of Helsinki. Consent in relation to the Data Protection Act 1998 and (where applicable) the Human Tissue Act 2004 has been obtained the relevant UK Biobank participants prior to enrollment. The Resource is available to all bona fide researchers for all types of health-related research that is in the public interest, and all applications to use the Resource are checked to ensure that research proposals are consistent with the Resource’s Access Procedures, the Ethics & Governance Framework and the consent that was provided by the participants.

### Genotyping and quality control

All participants were genotyped for approximately 800 000 genetic variants using the UK Biobank Axiom Array® or the highly similar UK BiLEVE Axiom® Array from Affymetrix (now Thermo Fisher Scientific Inc.), described elsewhere (42). Around 3% of participants did not undergo genotyping as insufficient DNA was extracted from blood samples, and fewer than 5% of genotyped markers exhibited sub-optimal quality and/or complex clustering patterns so were excluded from the data release (42). DNA microarray technology has been shown to be highly concordant with whole exome sequencing data even for rarer variants (43). We obtained genotyping data for all non-synonymous *CFI* rare variants represented in the UK Biobank DNA microarray (Table 1, Supplementary Material, Table S4). We also retrieved genotyping data for CFH(NM_000186):c.1336+892A>G, an intron variant selected as a proxy for *CFH* p.Y402H given its close (1 kb) proximity and high linkage disequilibrium (*r*^2^ = 0.99) (44), and ARMS2(NM_001099667):c.205G>T(p.A69S), contained within the common *ARMS2/HTRA1* AMD risk haplotype (45).

### Image storage and segmentation

All OCT images were stored as .fds and .fda files within a central repository at Advanced Research Computing, University of Oxford, UK, via remote login. The Topcon Advanced Boundary Segmentation (TABS™) segmentation software (Version 1.6.1.1, Topcon) was used to automatically segment the retinal surfaces as previously described (20,21). Internal limiting membrane–RPE thickness bands were calculated for both eyes and the mean of these derived; their outer limit is delineated by the photoreceptor–RPE interface. RPE–BM bands were calculated for one eye at random. The inner limit of the RPE–BM measure corresponds to the photoreceptor outer segment boundary, and its outer limit corresponds to the BM–choroid boundary. As the automated segmentation algorithm places the inner and outer boundaries of the RPE–BM complex on their respective hyporeflective edges, this results in a digital overestimation of overall RPE–BM thickness by 3.5 μm (21). We accessed these data through the UK Biobank returns catalogue.

### Diagnostic criteria

The linkage of all UK Biobank participants to health-related records allowed us to investigate the effect of genotype on AMD diagnosis. In the absence of robust mapping to AMD within the resource, we used the four-digit codes of the International Classification of Disease (ICD-10 and ICD-9) to identify individuals who received a diagnosis of degeneration of the macula and posterior pole of retina (H35.3 and 362.5, respectively) from hospital inpatient data and death registry data. We did not use self-reported estimates for AMD as the condition is often asymptomatic in its early stages, thus the accuracy of this measure is likely to be poorer with a greater underestimation of prevalence. We also obtained outcome data on MPGN and TMA in this manner.

### Inclusion and exclusion criteria

Participants who had undergone eye surgery less than 4 weeks ago were excluded from all ocular measurements. Subjects were excluded from segmentation analysis if they had poor OCT signal strength, poor image quality, poor centration certainty, missing thickness values from any ETDRS macular subfields, refractive error > +6 diopters (D) or < −6 D, visual acuity worse than 0.1 logMAR, IOPg ≥ 22 or  ≤ 5 mmHg or a self-reported history of ocular illness, diabetes or neurodegenerative disease—as previously defined (20,21). Additionally, participants with a health record diagnosis of AMD were excluded from analysis of RPE and retinal macular thickness associations with age as the timescale. Furthermore, participants with missing genotype information at our selected SNPs were withdrawn from their respective macular thickness and AMD association analyses. Finally, subjects who withdrew their consent were excluded from all downstream analyses. No exclusion criteria other than participant withdrawal were applied in the investigation of genotype association with AMD diagnosis.

### Statistical analyses

We performed statistical analyses using R (Version 4.0.5, Released 2021, The R Foundation for Statistical Computing Platform) and Minitab 17 Statistical Software (Version 18, Released 2017, Minitab Inc, State College, PA). We used the following aggregate terms for our analyses: *CFI* Type 1 RV (p.G119R, p.G287R, p.A240G, p.I357M, p.H418L, p.G162D, p.A431T, p.R474X, c.772 + 1G > T, p.P50A or p.G188V) and *CFI* VUS (p.G261D, p.R406H, p.K441R, or p.R345Q), in line with previous analyses of patient samples and/or recombinant protein (3,5–7,18,46–49) (Supplementary Material, Table S4). Although we identified carriers of *CFI* p.G188V, c.772 + 1G > T, p.A431T and p.R345Q with ICD-10 and ICD-9 diagnoses, none had available OCT-derived retina and RPE thickness metrics. *CFI* p.I340T is not reported in our analyses as it is the only known type 2 RV represented in the UK Biobank. Only 5% of participants had missing genetic data, which was due to instrumentation failure at the time of measurement rather than the demographics or allele combinations being interrogated. Therefore, we used a complete-case approach to subset the population by *CFI* variant functional type, i.e. removing any participants with missing *CFI* genotype data from downstream analyses.

We used univariable linear regression to compare the relationship of genotype to RPE and retinal thickness for each position on the ETDRS macular map, adjusting for multiple testing in subgroup analyses using Tukey multiple comparisons of means with 95% family-wise confidence level (Supplementary Material, Appendix 1). To further interrogate this relationship, we used stepwise (bidirectional) multivariable linear regression (*α* to enter = 0.05, *α* to remove = 0.1) using the covariates of *CFI* type 1 RV, *CFI* VUS, *CFH* p.Y402H and *ARMS2* p.A69S carrier status—as well as age, gender, ethnicity, smoking status, Townsend index, refraction, IOPg and SBP, which have previously been associated with both RPE and retinal thickness at the macula. We also included BMI among the potential covariates for the derivation of retinal thickness as a proxy for height, as the former was available for a 10-fold greater number of participants.

This stepwise approach encompasses the spatial variation in strength of association between macular thickness and clinical predictors, which has previously been reported (40). We tested our models for the assumptions of linear relationship, homogeneity of variance, multicollinearity, influential observations and normality of residuals, all of which supported our approach (Supplementary Material, Fig. S1). Where the predefined covariates did not reach significance to enter our stepwise model, we repeated the regression analysis including all terms to derive coefficients and significance levels, e.g. for individual genetic variants (Supplementary Material, Appendix 1). Finally, we used simple slopes analysis for two-way interactions to explore whether the relationship between mean macular thickness and age is significant at a particular value of the genotype (i.e. our moderator).

We undertook multilevel (mixed effects) logistic regression using maximum likelihood estimation (glm function in R) to investigate the relationship between each genotype and odds of AMD diagnosis adjusted for age, gender, ethnicity, smoking status and genotype and by selected genotypes as fixed effects, and for site of recruitment as a random effect to account for correlations between AMD risk and unobserved heterogeneity such as unmapped genetic variation that may segregate regionally. Finally, we examined the interactions between *CFI* type 1 RV, *CFI* VUS, *CFH* p.Y402H and *ARMS2* p.A69S genotypes on AMD diagnosis using a multivariable logistic regression model with age, gender, ethnicity, smoking status and genotype as independent variables, and the products of selected genotypes as interactive terms.

Our logistic regression model achieved favorable model fitting criteria, as demonstrated by the successful reduction of AIC with the sequential inclusion of covariables, a C-statistic of 0.75 indicating good-to-strong model discrimination, and non-significance of Hosmer–Lemeshow testing indicating that our model performs equally well across the range of probabilities of AMD (Supplementary Material, Table S5). Additionally, our model estimations and inferences are equivalent using fitted using maximum likelihood estimation, mean bias-reducing adjusted score and median bias-reduction adjusted score equations (Supplementary Material, Table S6), indicating that our model is robust to case–control and/or covariable imbalances (50).

For calculation of an empirical kinship matrix within RVTESTS, we used the Balding–Nicols method based on 42 757 genome-wide SNPs chosen to be in approximate equilibrium. For sensitivity analyses using the regenie package, we reduced the dimension of genetic data using ridge regression applied to blocks of 259 051 genome-wide SNPs exhibiting low levels of linkage equilibrium, and then combined the resulting predictors using a second round of linear or logistic ridge regression to produce an overall prediction for each trait. As a further sensitivity check, we also repeated the RVTESTS and regenie analyses removing all UK Biobank individuals that had been flagged by UK Biobank as being ‘non-white’.

For RVTESTS, which mean imputes any missing covariates to the mean, we included as covariates age, gender, smoking status, Townsend index, IOP, refraction, SBP and genotypes at *CFH* p.Y402H and *ARMS2* p.A69S (both coded as two-level factors). For regenie, which requires that all covariates be measured in all individuals, we included as covariates age, gender, smoking status and genotypes at *CFH* p.Y402H and *ARMS2* p.A69S (both coded as two-level factors). Prior to undertaking these analyses, we also excluded samples flagged as failing the centrally performed quality controls for heterozygosity, and those flagged as having a mismatch between self-reported and genotype-derived gender or potential sex chromosome aneuploidy. We did not exclude participants with missing *CFI* genotypes from our sensitivity analyses.

## Supplementary Material

Supplemental_Appendix_1_ddac060

Supplemental_Appendix_2_ddac060

Supplemental_Figure_1_ddac060

Supplemental_Figure_2_ddac060

Supplemental_Figure_3_ddac060

Supplemental_Figure_4_ddac060

Supplemental_Table_1_ddac060

Supplemental_Table_2_ddac060

Supplemental_Table_3_ddac060

Supplemental_Table_4_ddac060

Supplemental_Table_5_ddac060

Supplemental_Table_6_ddac060
