# Supplementary material for: Rare complement factor I variants associated with reduced macular thickness and age-related macular degeneration in the UK Biobank
Source: Hum Mol Genet. 2022 Mar 14;31(16):2678–92. doi: 10.1093/hmg/ddac060 (PMC9402241; doi:10.1093/hmg/ddac060)
Supplement: Supplemental_Appendix_1_ddac060 [file supplemental_appendix_1_ddac060.pdf]

Supplemental Appendix 1

The following tables present the results of association analyses for grouped and individual genotypes apropos optical coherence tomography (OCT)-derived retinal pigment epithelium (RPE) and retinal macular thickness metrics. Individual rare variants (RVs) in the complement factor I gene (*CFI*) are categorized into type 1 or variant of uncertain significance (VUS) groups based on their association with low or normal, respectively, Factor I levels. We also report regression coefficients for carriers of any *CFI* RV that included the type 1 RVs and VUS, as well as p.I340T (a type 2 RV, that is associated with normal Factor I levels but impaired function), and *CFI* p.T62S, for which there is insufficient information regarding its impact on Factor I production or function and, therefore, its categorization as type 1, type 2, or benign. Significant differences are indicated by * for $P < 0.05$, ** for $P < 0.01$, and *** for $P < 0.001$.

Statistical considerations: Minimum observations indicate the number of variants available for comparison against 34,458 participants with validated OCT-derived RPE thickness metrics and 32,057 participants with validated OCT-derived retinal thickness metrics. Other than two p.G261D homozygotes with RPE thickness metrics and two p.R406H homozygotes with retinal thickness metrics, the remaining *CFI* RVs/VUS included in our analyses were heterozygotes. We adjusted univariable linear regression analyses for multiple corrections using Tukey multiple comparisons of means with 95% family-wise confidence level. We used a stepwise approach with bidirectional elimination (α to enter = 0.05, α to remove = 0.1) and the covariates of *CFI* type 1 RV, *CFI* VUS, complement factor H gene (*CFH)* p.Y402H, and age-related maculopathy susceptibility protein 2 gene *(ARMS2)* p.A69S carrier status as well as age, gender, ethnicity, smoking status, refraction, Goldmann-corrected intraocular pressure, and systolic blood pressure for our multivariable linear regression analyses. For multivariable retinal thickness analyses, body mass index was also included as a covariate as a proxy for height. For *CFH* p.Y402H and *ARMS2* p.A69S, wild-type (WT) vs. heterozygous and homozygous comparisons were included in our multivariable analyses, but

heterozygous/homozygous comparisons were only possible for univariable analyses. To derive unstandardized coefficients for individual variants, we replaced the *CFI* type 1 RV and *CFI* VUS covariates in our stepwise models with these. To derive unstandardized coefficients for genotypes which did not reach the threshold significance to enter our stepwise models, we used a forced-entry hierarchical linear regression including all demographic/clinical predictors and all individual variants. Regression coefficients for clinical and demographic covariates are not presented. The adjusted R-squared for all models ranged from 4–10%.

Mean RPE thickness

**Genotype**	Minimum observations	*P* value (univariable)	*P* value (multivariable)	Coefficient, µm (multivariable)	95% confidence interval, µm (multivariable)
***CFH* p.Y402H** **heterozygous**	15438	<0.001 ***	<0.001 ***	−0.25	(−0.31, −0.18)
***CFH* p.Y402H** **homozygous**	4757	<0.001 ***	<0.001 ***	−0.52	(−0.62, −0.42)
***CFH* p.Y402H** **homozygous vs heterozygous**	4757	<0.001 ***	NA	NA	NA
***ARMS2* p.A69S** **heterozygous**	11409	1.00	0.80	−0.01	(−0.08, 0.06)
***ARMS2* p.A69S** **homozygote**	1627	0.53	0.12	−0.12	(−0.27, 0.03)
***ARMS2* p.A69S** **homozygous vs heterozygous**	1627	0.56	NA	NA	NA
***CFI* Type 1 RV**	85	0.001 **	0.002 **	−1.01	(−1.66, −0.37)
***CFI* VUS**	486	0.22	0.28	−0.15	(−0.41, 0.12)
***CFI* any RV**	582	0.02 *	0.03 *	−0.27	(−0.51, −0.03)
***CFI* p.G119R**	57	0.03 *	0.01 *	−0.96	(−1.72, −0.20)
***CFI* p.G287R**	14	0.01 *	0.02 *	−1.97	(−3.67, −0.26)
***CFI* p.A240G**	4	0.06	0.14	−2.06	(−4.76, 0.64)
***CFI* p.H418L**	3	0.60	0.12	3.02	(−0.79, 6.84)
***CFI* p.R474X**	2	0.24	0.24	−2.28	(−6.09, 1.54)
***CFI* p.P50A**	2	0.97	0.84	0.38	(−3.43, 4.20)
***CFI* p.I357M**	2	0.85	0.38	2.43	(−2.97, 7.83)
***CFI* p.G162D**	1	0.43	NA	NA	NA
***CFI* p.I340T**	8	0.53	0.40	0.82	(−1.09, 2.73)
***CFI* p.R406H**	193	0.55	0.42	0.18	(−0.25, 0.61)
***CFI* p.G261D**	190	0.05	0.04 *	−0.42	(−0.83, −0.02)
***CFI* p.K441R**	104	0.22	0.67	−0.12	(−0.68, 0.43)
***CFI* p.T62S**	3	0.81	0.70	−0.75	(−4.57, 3.07)

Central RPE thickness

Genotype	Minimum observations	*P* value (univariable)	*P* value (multivariable)	Coefficient, µm (multivariable)	95% confidence interval, µm (multivariable)
***CFH* p.Y402H** **heterozygous vs WT**	15438	<0.001 ***	<0.001 ***	−0.27	(−0.38, −0.16)
***CFH* p.Y402H** **homozygous vs WT**	4757	<0.001 ***	<0.001 ***	−0.55	(−0.71, −0.39)
***CFH* p.Y402H** **homozygous vs heterozygous**	4757	<0.001 ***	NA	NA	NA
***ARMS2* p.A69S** **heterozygous vs WT**	11409	0.20	0.10	0.09	(−0.02, −0.20)
***ARMS2* p.A69S** **homozygous vs WT**	1627	0.70	0.62	0.06	(−0.18, 0.30)
***ARMS2* p.A69S** **homozygous vs heterozygous**	1627	1.00	NA	NA	NA
***CFI* Type 1 RV**	85	0.28	0.72	−0.20	(−1.24, 0.85)
***CFI* VUS**	486	0.70	0.85	−0.04	(−0.47, −0.39)
***CFI* any RV**	582	0.97	0.89	−0.03	(−0.42, 0.37)
***CFI* p.G119R**	57	0.52	0.98	−0.02	(−1.25, 1.22)
***CFI* p.G287R**	14	0.27	0.34	−1.37	(−4.16, 1.42)
***CFI* p.A240G**	4	0.51	0.59	−1.22	(−0.56, 3.20)
***CFI* p.H418L**	3	0.62	0.09	5.42	(−0.82, 11.67)
***CFI* p.R474X**	2	0.60	0.78	−0.90	(−7.14, 5.34)
***CFI* p.P50A**	2	0.89	0.77	0.91	(−5.33, 7.16)
***CFI* p.I357M**	2	0.80	0.60	−2.38	(−11.21, 6.45)
***CFI* p.G162D**	1	0.74	NA	NA	NA
***CFI* p.I340T**	8	0.51	0.56	0.93	(−2.20, 4.05)
***CFI* p.R406H**	193	0.55	0.29	0.38	(−0.33, 1.08)
***CFI* p.G261D**	190	0.49	0.45	−0.26	(−7.53, 4.95)
***CFI* p.K441R**	104	0.39	0.73	−0.16	(−1.07, 0.75)
***CFI* p.T62S**	3	0.45	0.69	−1.29	(−7.53, 4.95)

Inner RPE thickness

**Genotype**	Minimum observations	*P* value (univariable)	*P* value (multivariable)	Coefficient, µm (multivariable)	95% confidence interval, µm (multivariable)
***CFH* p.Y402H** heterozygous vs WT	15438	<0.001 ***	<0.001 ***	−0.22	(−0.31, −0.13)
***CFH* p.Y402H** homozygous vs WT	4757	<0.001 ***	<0.001 ***	−0.48	(−0.60, −0.35)
***CFH* p.Y402H** homozygous vs heterozygous	4757	<0.001 ***	NA	NA	NA
***ARMS2* p.A69S** heterozygous vs WT	11409	0.99	0.73	−0.02	(−0.10, 0.07)
***ARMS2* p.A69S** homozygous vs WT	1627	0.98	0.49	−0.07	(−1.66, −0.00)
***ARMS2* p.A69S** homozygous vs heterozygous	1627	0.99	NA	NA	NA
***CFI* Type 1 RV**	85	0.02 *	0.049 *	−0.83	(−1.66, −0.00)
***CFI* VUS**	486	0.86	0.84	−0.04	(−0.38, 0.30)
***CFI* any RV**	582	0.46	0.38	−0.14	(−0.45, 0.17)
***CFI* p.G119R**	57	0.10	0.10	−0.82	(−1.79, 0.16)
***CFI* p.G287R**	14	0.14	0.18	−1.51	(−3.71, 0.70)
***CFI* p.A240G**	4	0.32	0.45	−1.33	(−4.82, 2.15)
***CFI* p.H418L**	3	0.83	0.24	2.93	(−2.00, 7.86)
***CFI* p.R474X**	2	0.21	0.25	−2.90	(−7.83, 2.03)
***CFI* p.P50A**	2	0.97	0.73	0.86	(−4.08, 5.79)
***CFI* p.I357M**	2	0.84	0.52	2.28	(−4.70, 9.25)
***CFI* p.G162D**	1	0.49	NA	NA	NA
***CFI* p.I340T**	8	0.79	0.71	0.46	(−2.00, 2.93)
***CFI* p.R406H**	193	0.06	0.15	0.41	(−0.14, 0.96)
***CFI* p.G261D**	190	0.28	0.22	−0.33	(−0.86, 0.20)
***CFI* p.K441R**	104	0.24	0.70	−0.14	(−0.86, 0.58)
***CFI* p.T62S**	3	0.66	0.48	−1.76	(−6.69, 3.17)

Outer RPE thickness

Genotype	Minimum observations	*P* value (univariable)	*P* value (multivariable)	Coefficient, µm (multivariable)	95% confidence interval, µm (multivariable)
***CFH* p.Y402H** **heterozygous vs WT**	15438	<0.001 ***	<0.001 ***	−0.25	(−0.32, −0.19)
***CFH* p.Y402H** **homozygous vs WT**	4757	<0.001 ***	<0.001 ***	−0.53	(−0.63, −0.44)
***CFH* p.Y402H** **homozygous vs heterozygous**	4757	<0.001 ***	NA	NA	NA
***ARMS2* p.A69S** **heterozygous vs WT**	11409	0.99	0.75	−0.01	(−0.08, −0.05)
***ARMS2* p.A69S** **homozygous vs WT**	1627	0.31	0.05	−0.14	(−0.28, 0.00)
***ARMS2* p.A69S** **homozygous vs heterozygous**	1627	0.36	NA	NA	NA
***CFI* Type 1 RV**	85	<0.001 ***	0.001 **	−1.10	(−1.72, −0.47)
***CFI* VUS**	486	0.07	0.16	−0.18	(−0.44, 0.07)
***CFI* any RV**	582	0.003 **	0.01 *	−0.30	(−0.54, −0.07)
***CFI* p.G119R**	57	0.02 *	0.006 **	−1.03	(−1.76, −0.30)
***CFI* p.G287R**	14	0.004 **	0.01 *	−2.12	(−3.78, −0.47)
***CFI* p.A240G**	4	0.03 *	0.09	−2.30	(−4.92, 0.32)
***CFI* p.H418L**	3	0.54	0.12	2.96	(−0.74, 6.66)
***CFI* p.R474X**	2	0.28	0.26	−2.14	(−5.84, 1.56)
***CFI* p.P50A**	2	0.94	0.91	0.22	(−3.48, 3.92)
***CFI* p.I357M**	2	0.86	0.32	2.66	(−2.57, 7.90)
***CFI* p.G162D**	1	0.42	NA	NA	NA
***CFI* p.I340T**	8	0.48	0.33	0.92	(−0.93, 2.77)
***CFI* p.R406H**	193	0.77	0.63	0.10	(−0.31, 0.52)
***CFI* p.G261D**	190	0.03 *	0.02 *	−0.46	(−0.86, −0.06)
***CFI* p.K441R**	104	0.24	0.67	−0.12	(−0.65, 0.42)
***CFI* p.T62S**	3	0.91	0.82	−0.43	(−4.13, 3.27)

Temporal RPE thickness

**Genotype**	Minimum observations	*P* value (univariable)	*P* value (multivariable)	Coefficient, µm (multivariable)	95% confidence interval, µm (multivariable)
***CFH* p.Y402H** **heterozygous vs WT**	15438	<0.001 ***	<0.001 ***	−0.17	(−0.26, −0.08)
***CFH* p.Y402H** **homozygous vs WT**	4757	<0.001 ***	<0.001 ***	−0.39	(−0.51, −0.27)
***CFH* p.Y402H** **homozygous vs heterozygous**	4757	<0.001 ***	NA	NA	NA
***ARMS2* p.A69S** **heterozygous vs WT**	11409	0.85	0.54	0.027	(−0.06, 0.11)
***ARMS2* p.A69S** **homozygous vs WT**	1627	0.95	0.87	−0.02	(−0.20, 0.17)
***ARMS2* p.A69S** **homozygous vs heterozygous**	1627	1.00	NA	NA	NA
***CFI* Type 1 RV**	85	0.01 *	0.02 *	−0.96	(−1.77, −0.14)
***CFI* VUS**	486	0.57	0.63	−0.08	(−0.42, 0.25)
***CFI* any RV**	582	0.14	0.23	−0.19	(−0.50, 0.12)
***CFI* p.G119R**	57	0.11	0.07	−0.88	(−1.84, 0.08)
***CFI* p.G287R**	14	0.02 *	0.04 *	−2.28	(−4.45, −0.12)
***CFI* p.A240G**	4	0.35	0.56	−1.02	(−1.94, 7.75)
***CFI* p.H418L**	3	0.80	0.24	2.90	(−1.94, 7.75)
***CFI* p.R474X**	2	0.22	0.23	−2.98	(−7.82, 1.87)
***CFI* p.P50A**	2	0.63	0.93	−0.22	(−5.07, 4.62)
***CFI* p.I357M**	2	0.87	0.21	4.37	(−2.48, 11.22)
***CFI* p.G162D**	1	0.20	NA	NA	NA
***CFI* p.I340T**	8	0.76	0.56	0.72	(−1.70, 3.14)
***CFI* p.R406H**	193	0.48	0.33	0.27	(−0.27, 0.82)
***CFI* p.G261D**	190	0.12	0.10	−0.44	(−0.96, 0.08)
***CFI* p.K441R**	104	0.60	0.80	0.09	(−0.61, 0.90)
***CFI* p.T62S**	3	0.71	0.62	−1.24	(−6.08, 3.61)

Superior RPE thickness

**Genotype**	Minimum observations	*P* value (univariable)	*P* value (multivariable)	Coefficient, µm (multivariable)	95% confidence interval, µm (multivariable)
***CFH* p.Y402H** **heterozygous vs WT**	15438	<0.001 ***	<0.001 ***	−0.27	(−0.35, −0.19)
***CFH* p.Y402H** **homozygous vs WT**	4757	<0.001 ***	<0.001 ***	−0.57	(−0.68, −0.46)
***CFH* p.Y402H** **homozygous vs heterozygous**	4757	<0.001 ***	NA	NA	NA
***ARMS2* p.A69S** **heterozygous vs WT**	11409	0.41	0.09	−0.06	(−0.14, 0.01)
***ARMS2* p.A69S** **homozygous vs WT**	1627	0.01 *	0.002 **	−0.26	(−0.42, −0.10)
***ARMS2* p.A69S** **homozygous vs heterozygous**	1627	0.06	NA	NA	NA
***CFI* Type 1 RV**	85	<0.001 ***	0.004 **	−1.04	(−1.76, −0.32)
***CFI* VUS**	486	0.40	0.37	−0.14	(−0.43, 0.16)
***CFI* any RV**	582	0.04 *	0.04 *	−0.28	(−0.54, −0.01)
***CFI* p.G119R**	57	0.005 **	0.01 *	−1.12	(−1.96, −0.27)
***CFI* p.G287R**	14	0.02 *	0.05 *	−1.92	(−3.82, −0.01)
***CFI* p.A240G**	4	0.19	0.29	−1.63	(−4.64, 1.38)
***CFI* p.H418L**	3	0.44	0.07	4.00	(−0.26, 8.26)
***CFI* p.R474X**	2	0.43	0.52	−1.41	(−5.67, 2.85)
***CFI* p.P50A**	2	0.68	0.48	1.54	(−2.72, 5.80)
***CFI* p.I357M**	2	0.48	0.96	−0.15	(−6.17, 5.88)
***CFI* p.G162D**	1	0.31	NA	NA	NA
***CFI* p.I340T**	8	0.72	0.65	0.49	(1.64, 2.62)
***CFI* p.R406H**	193	0.22	0.44	0.189	(−0.29, 0.67)
***CFI* p.G261D**	190	0.24	0.30	−0.24	(−0.70, 0.22)
***CFI* p.K441R**	104	0.02 *	0.14	−0.46	(−1.08, 0.16)
***CFI* p.T62S**	3	0.82	0.64	−1.03	(−5.29, 3.23)

Nasal RPE thickness

**Genotype**	Minimum observations	*P* value (univariable)	*P* value (multivariable)	Coefficient, µm (multivariable)	95% confidence interval, µm (multivariable)
***CFH* p.Y402H** **heterozygous vs WT**	15438	<0.001 ***	<0.001 ***	−0.22	(−0.31, −0.14)
***CFH* p.Y402H** **homozygous vs WT**	4757	<0.001 ***	<0.001 ***	−0.48	(−0.61, −0.36)
***CFH* p.Y402H** **homozygous vs heterozygous**	4757	<0.001 ***	NA	NA	NA
***ARMS2* p.A69S** **heterozygous vs WT**	11409	0.41	0.22	0.05	(−0.03, 0.14)
***ARMS2* p.A69S** **homozygous vs WT**	1627	0.59	0.59	0.05	(−0.14, 0.24)
***ARMS2* p.A69S** **homozygous vs heterozygous**	1627	0.92	NA	NA	NA
***CFI* Type 1 RV**	85	0.006 **	0.003 **	−1.23	(−2.05, −0.41)
***CFI* VUS**	486	0.59	0.61	−0.09	(−0.43, 0.25)
***CFI* any RV**	582	0.14	0.15	−0.23	(−0.54, 0.08)
***CFI* p.G119R**	57	0.06	0.02 *	−1.16	(−2.13, −0.19)
***CFI* p.G287R**	14	0.08	0.06	−2.08	(−4.27, 0.12)
***CFI* p.A240G**	4	0.10	0.20	−2.26	(−5.73, 1.21)
***CFI* p.H418L**	3	0.91	0.39	2.14	(−2.76, 7.04)
***CFI* p.R474X**	2	0.23	0.20	−3.23	(−8.13, 1.67)
***CFI* p.P50A**	2	0.90	0.95	−0.17	(−5.07, 4.74)
***CFI* p.I357M**	2	0.71	0.09	6.00	(−0.94, 12.93)
***CFI* p.G162D**	1	0.69	NA	NA	NA
***CFI* p.I340T**	8	0.47	0.36	1.14	(−1.32, 3.59)
***CFI* p.R406H**	193	0.56	0.22	0.346	(−0.20, 0.90)
***CFI* p.G261D**	190	0.10	0.04 *	−0.56	(−1.08, −0.03)
***CFI* p.K441R**	104	0.81	0.76	0.113	(−0.60, 0.83)
***CFI* p.T62S**	3	0.94	0.62	−1.24	(−6.15, 3.66)

Inferior RPE thickness

**Genotype**	Minimum observations	*P* value (univariable)	*P* value (multivariable)	Coefficient, µm (multivariable)	95% confidence interval, µm (multivariable)
CFH **p.Y402H heterozygous vs WT**	15438	<0.001 ***	<0.001 ***	−0.28	(−0.35, −0.20)
CFH **p.Y402H homozygous vs WT**	4757	<0.001 ***	<0.001 ***	−0.58	(−0.68, −0.47)
CFH **p.Y402H homozygous vs heterozygous**	4757	<0.001 ***	NA	NA	NA
ARMS2 **p.A69S heterozygous vs WT**	11409	0.40	0.07	−0.07	(−0.14, 0.01)
ARMS2 **p.A69S homozygous vs WT**	1627	0.24	0.04 *	−0.17	(−0.33, −0.01)
ARMS2 **p.A69S homozygous vs heterozygous**	1627	0.57	NA	NA	NA
CFI **Type 1 RV**	85	0.04 *	0.09	−0.61	(−1.31, 0.09)
CFI **VUS**	486	0.53	0.38	−0.13	(−0.42, 0.16)
CFI **any RV**	582	0.16	0.16	−0.19	(−0.45, 0.08)
CFI **p.G119R**	57	0.29	0.24	−0.49	(−1.31, 0.33)
CFI **p.G287R**	14	0.11	0.30	−0.99	(−2.85, 0.87)
CFI **p.A240G**	4	0.06	0.12	−2.36	(−5.29, 0.58)
CFI **p.H418L**	3	0.70	0.20	2.74	(−1.41, 6.89)
CFI **p.R474X**	2	0.21	0.24	−2.47	(−6.62, 1.69)
CFI **p.P50A**	2	0.81	0.64	1.00	(−3.15, 5.16)
CFI **p.I357M**	2	0.48	0.91	−0.34	(−6.22, 5.53)
CFI **p.G162D**	1	0.96	NA	NA	NA
CFI **p.I340T**	8	0.75	0.69	0.42	(−1.66, 2.49)
CFI **p.R406H**	193	0.19	0.36	0.22	(−0.25, 0.68)
CFI **p.G261D**	190	0.13	0.13	−0.34	(−0.79, 0.10)
CFI **p.K441R**	104	0.12	0.41	−0.25	(−0.86, 0.35)
CFI **p.T62S**	3	0.62	0.68	−0.87	(−5.03, 3.28)

Inner temporal RPE thickness

**Genotype**	Minimum observations	*P* value (univariable)	*P* value (multivariable)	Coefficient, µm (multivariable)	95% confidence interval, µm (multivariable)
***CFH* p.Y402H** heterozygous vs WT	15438	0.004 **	0.004 **	−0.15	(−0.25, −0.05)
***CFH* p.Y402H** homozygous vs WT	4757	<0.001 ***	<0.001 ***	−0.33	(−0.47, −0.19)
***CFH* p.Y402H** homozygous vs heterozygous	4757	0.01 *	NA	NA	NA
***ARMS2* p.A69S** heterozygous vs WT	11409	0.73	0.40	0.04	(−0.06, 0.14)
***ARMS2* p.A69S** homozygous vs WT	1627	0.60	0.56	0.07	(−0.15, 0.28)
***ARMS2* p.A69S** homozygous vs heterozygous	1627	0.82	NA	NA	NA
***CFI* Type 1 RV**	85	0.08	0.08	−0.84	(−1.78, 0.10)
***CFI* VUS**	486	0.83	0.87	−0.03	(−0.42, 0.35)
***CFI* any RV**	582	0.38	0.48	−0.13	(−0.48, 0.23)
***CFI* p.G119R**	57	0.17	0.12	−0.88	(−1.99, 0.23)
***CFI* p.G287R**	14	0.10	0.10	−2.10	(−4.61, 0.40)
***CFI* p.A240G**	4	0.54	0.73	−0.71	(−4.67, 3.26)
***CFI* p.H418L**	3	0.50	0.14	4.23	(−1.37, 9.84)
***CFI* p.R474X**	2	0.26	0.28	−3.08	(−8.68, 2.52)
***CFI* p.P50A**	2	0.93	0.81	0.69	(−4.92, 6.30)
***CFI* p.I357M**	2	0.73	0.21	5.11	(−2.82, 13.03)
***CFI* p.G162D**	1	0.14	NA	NA	NA
***CFI* p.I340T**	8	0.70	0.55	0.85	(−1.95, 3.65)
***CFI* p.R406H**	193	0.52	0.27	0.36	(−0.27, 0.99)
***CFI* p.G261D**	190	0.30	0.21	−0.39	(−0.99, 0.22)
***CFI* p.K441R**	104	0.67	0.78	0.12	(−0.70, 0.93)
***CFI* p.T62S**	3	0.56	0.37	−2.58	(−8.19, 3.02)

Inner superior RPE thickness

**Genotype**	Minimum observations	*P* value (univariable)	*P* value (multivariable)	Coefficient, µm (multivariable)	95% confidence interval, µm (multivariable)
***CFH* p.Y402H** heterozygous vs WT	15438	<0.001 ***	<0.001 ***	−0.26	(−0.36, −0.16)
***CFH* p.Y402H** homozygous vs WT	4757	<0.001 ***	<0.001 ***	−0.58	(−0.71, −0.44)
***CFH* p.Y402H** homozygous vs heterozygous	4757	<0.001 ***	NA	NA	NA
***ARMS2* p.A69S** heterozygous vs WT	11409	0.59	0.18	−0.07	(−0.16, 0.03)
***ARMS2* p.A69S** homozygous vs WT	1627	0.07	0.02 *	−0.25	(−0.46, −0.04)
***ARMS2* p.A69S** homozygous vs heterozygous	1627	0.20	NA	NA	NA
***CFI* Type 1 RV**	85	0.006 **	0.04 *	−0.97	(−1.89, −0.05)
***CFI* VUS**	486	0.99	0.78	−0.05	(−0.43, 0.32)
***CFI* any RV**	582	0.27	0.28	−0.19	(−0.54, 0.15)
***CFI* p.G119R**	57	0.02 *	0.05	−1.07	(−2.16, 0.01)
***CFI* p.G287R**	14	0.08	0.12	−1.94	(−4.38, 0.50)
***CFI* p.A240G**	4	0.51	0.62	−0.97	(−4.82, 2.89)
***CFI* p.H418L**	3	0.54	0.08	4.87	(−0.59, 10.32)
***CFI* p.R474X**	2	0.41	0.53	−1.75	(−7.20, 3.71)
***CFI* p.P50A**	2	0.72	0.53	1.77	(−3.69, 7.23)
***CFI* p.I357M**	2	0.53	0.89	−0.56	(−8.27, 7.16)
***CFI* p.G162D**	1	0.41	NA	NA	NA
***CFI* p.I340T**	8	0.74	0.76	0.42	(−2.31, 3.15)
***CFI* p.R406H**	193	0.07	0.23	0.37	(−0.24, 0.99)
***CFI* p.G261D**	190	0.53	0.59	−0.16	(−0.75, 0.42)
***CFI* p.K441R**	104	0.03 *	0.20	−0.52	(−1.32, 0.27)
***CFI* p.T62S**	3	0.49	0.40	−2.33	(−7.78, 3.13)

Inner nasal RPE thickness

**Genotype**	Minimum observations	*P* value (univariable)	*P* value (multivariable)	Coefficient, µm (multivariable)	95% confidence interval, µm (multivariable)
***CFH* p.Y402H** **heterozygous vs WT**	15438	<0.001 ***	<0.001 ***	−0.20	(−0.30, −0.10)
***CFH* p.Y402H** **homozygous vs WT**	4757	<0.001 ***	<0.001 ***	−0.45	(−0.60, −0.31)
***CFH* p.Y402H** **homozygous vs heterozygous**	4757	0.002 **	NA	NA	NA
***ARMS2* p.A69S** **heterozygous vs WT**	11409	0.88	0.68	0.02	(−0.08, 0.12)
***ARMS2* p.A69S** **homozygous vs WT**	1627	0.57	0.65	0.05	(−0.17, 0.27)
***ARMS2* p.A69S** **homozygous vs heterozygous**	1627	0.72	NA	NA	NA
***CFI* Type 1 RV**	85	0.01 *	0.02 *	−1.17	(−2.13, −0.21)
***CFI* VUS**	486	0.58	0.92	0.02	(−0.37, 0.41)
***CFI* any RV**	582	0.70	0.48	−0.13	(−0.49, 0.23)
***CFI* p.G119R**	57	0.08	0.05	−1.12	(−2.24, 0.01)
***CFI* p.G287R**	14	0.18	0.17	−1.78	(−4.32, 0.77)
***CFI* p.A240G**	4	0.23	0.33	−2.01	(−6.04, 2.02)
***CFI* p.H418L**	3	0.57	0.97	0.13	(−5.57, 5.83)
***CFI* p.R474X**	2	0.26	0.25	−3.32	(−9.02, 2.37)
***CFI* p.P50A**	2	0.80	0.90	−0.38	(−6.08, 5.32)
***CFI* p.I357M**	2	0.52	0.06	7.90	(−0.16, 15.95)
***CFI* p.G162D**	1	0.93	NA	NA	NA
***CFI* p.I340T**	8	0.75	0.64	0.67	(−2.18, 3.52)
***CFI* p.R406H**	193	0.07	0.10	0.53	(−0.11, 1.17)
***CFI* p.G261D**	190	0.27	0.14	−0.46	(−1.08, 0.15)
***CFI* p.K441R**	104	0.81	0.71	0.16	(−0.67, 0.98)
***CFI* p.T62S**	3	0.73	0.82	−0.68	(−6.37, 5.02)

Inner inferior RPE thickness

**Genotype**	Minimum observations	*P* value (univariable)	*P* value (multivariable)	Coefficient, µm (multivariable)	95% confidence interval, µm (multivariable)
***CFH* p.Y402H** **heterozygous vs WT**	15438	<0.001 ***	<0.001 ***	−0.27	(−0.37, −0.17)
***CFH* p.Y402H** **homozygous vs WT**	4757	<0.001 ***	<0.001 ***	−0.56	(−0.70, −0.42)
***CFH* p.Y402H** **homozygous vs heterozygous**	4757	<0.001 ***	NA	NA	NA
***ARMS2* p.A69S** **heterozygous vs WT**	11409	0.77	0.24	−0.06	(−0.16, 0.04)
***ARMS2* p.A69S** **homozygous vs WT**	1627	0.85	0.29	−0.12	(−0.33, 0.10)
***ARMS2* p.A69S** **homozygous vs heterozygous**	1627	0.97	NA	NA	NA
***CFI* Type 1 RV**	85	0.25	0.48	−0.33	(−1.26, 0.60)
***CFI* VUS**	486	0.78	0.69	−0.08	(−0.46, 0.31)
***CFI* any RV**	582	0.81	0.54	−0.11	(−0.46, 0.24)
***CFI* p.G119R**	57	0.68	0.73	−0.19	(−1.29, 0.91)
***CFI* p.G287R**	14	0.57	0.87	−0.20	(−2.68, 2.28)
***CFI* p.A240G**	4	0.29	0.41	−1.65	(−5.57, 2.27)
***CFI* p.H418L**	3	0.96	0.38	2.49	(−3.06, 8.03)
***CFI* p.R474X**	2	0.17	0.22	−3.44	(−8.98, 2.10)
***CFI* p.P50A**	2	0.89	0.64	1.34	(−4.20, 6.88)
***CFI* p.I357M**	2	0.27	0.40	−3.34	(−11.18, 4.50)
***CFI* p.G162D**	1	0.96	NA	NA	NA
***CFI* p.I340T**	8	0.92	0.95	−0.09	(−2.86, 2.68)
***CFI* p.R406H**	193	0.03 *	0.23	0.38	(−0.24, 1.00)
***CFI* p.G261D**	190	0.29	0.29	−0.32	(−0.92, 0.27)
***CFI* p.K441R**	104	0.17	0.46	−0.31	(−1.11, 0.50)
***CFI* p.T62S**	3	0.51	0.60	−1.47	(−8.98, 2.10)

Outer temporal RPE thickness

Genotype	Minimum observations	*P* value (univariable)	*P* value (multivariable)	Coefficient, μm (multivariable)	95% confidence interval, μm (multivariable)
***CFH* p.Y402H** **heterozygous vs WT**	15438	<0.001 ***	<0.001 ***	−0.19	(−0.27, −0.10)
***CFH* p.Y402H** **homozygous vs WT**	4757	<0.001 ***	<0.001 ***	−0.44	(−0.56, −0.32)
***CFH* p.Y402H** **homozygous vs heterozygous**	4757	<0.001 ***	NA	NA	NA
***ARMS2* p.A69S** **heterozygous vs WT**	11409	0.98	0.79	0.01	(−0.07, 0.09)
***ARMS2* p.A69S** **homozygous vs WT**	1627	0.87	0.30	−0.10	(−0.28, 0.08)
***ARMS2* p.A69S** **homozygous vs heterozygous**	1627	0.83	NA	NA	NA
***CFI* Type 1 RV**	85	0.002 **	0.007 **	−1.07	(−1.85, −0.29)
***CFI* VUS**	486	0.36	0.43	−0.13	(−0.45, 0.19)
***CFI* any RV**	582	0.05 *	0.10	−0.25	(−0.54, 0.05)
***CFI* p.G119R**	57	0.10	0.06	−0.89	(−1.812, 0.04)
***CFI* p.G287R**	14	0.005 **	0.02 *	−2.49	(−4.57, −0.40)
***CFI* p.A240G**	4	0.23	0.43	−1.34	(−4.64, 1.96)
***CFI* p.H418L**	3	0.78	0.51	1.58	(−3.09, 6.24)
***CFI* p.R474X**	2	0.23	0.23	−2.87	(−7.54, 1.79)
***CFI* p.P50A**	2	0.38	0.63	−1.14	(−5.80, 3.53)
***CFI* p.I357M**	2	0.94	0.28	3.63	(−2.97, 10.23)
***CFI* p.G162D**	1	0.37	NA	NA	NA
***CFI* p.I340T**	8	0.87	0.62	0.59	(−1.74, 2.92)
***CFI* p.R406H**	193	0.34	0.48	0.19	(−0.33, 0.71)
***CFI* p.G261D**	190	0.05 *	0.05	−0.50	(−1.00, −0.00)
***CFI* p.K441R**	104	0.55	0.85	0.07	(−0.61, 0.75)
***CFI* p.T62S**	3	0.94	0.97	0.11	(−4.56, 4.77)

Outer superior RPE thickness

**Genotype**	Minimum observations	*P* value (univariable)	*P* value (multivariable)	Coefficient, µm (multivariable)	95% confidence interval, µm (multivariable)
***CFH* p.Y402H** **heterozygous vs WT**	15438	<0.001 ***	<0.001 ***	−0.28	(−0.35, −0.21)
***CFH* p.Y402H** **homozygous vs WT**	4757	<0.001 ***	<0.001 ***	−0.56	(−0.66, −0.47)
***CFH* p.Y402H** **homozygous vs heterozygous**	4757	<0.001 ***	NA	NA	NA
***ARMS2* p.A69S** **heterozygous vs WT**	11409	0.31	0.06	−0.06	(−0.13, 0.00)
***ARMS2* p.A69S** **homozygous vs WT**	1627	0.003 **	<0.001 ***	−0.27	(−0.43, −0.13)
***ARMS2* p.A69S** **homozygous vs heterozygous**	1627	0.03 *	NA	NA	NA
***CFI* Type 1 RV**	85	<0.001 ***	0.001 **	−1.12	(−1.76, −0.47)
***CFI* VUS**	486	0.06	0.10	−0.22	(−0.48, 0.04)
***CFI* any RV**	582	0.002 **	0.003 **	−0.36	(−0.60, −0.12)
***CFI* p.G119R**	57	0.003 **	0.003 **	−1.16	(−1.91, −0.40)
***CFI* p.G287R**	14	0.01 *	0.03 *	−1.88	(−3.59, −0.17)
***CFI* p.A240G**	4	0.05 *	0.10	−2.30	(−5.00, 0.41)
***CFI* p.H418L**	3	0.40	0.11	3.14	(−0.69, 6.96)
***CFI* p.R474X**	2	0.56	0.58	−1.07	(−4.90, 2.76)
***CFI* p.P50A**	2	0.68	0.50	1.32	(−2.51, 5.14)
***CFI* p.I357M**	2	0.50	0.92	0.26	(−5.15, 5.67)
***CFI* p.G162D**	1	0.28	NA	NA	NA
***CFI* p.I340T**	8	0.73	0.57	0.56	(−1.36, 2.47)
***CFI* p.R406H**	193	0.72	0.98	0.01	(−0.42, 0.44)
***CFI* p.G261D**	190	0.08	0.13	−0.32	(−0.73, 0.09)
***CFI* p.K441R**	104	0.03	0.16	−0.40	(−0.96, 0.16)
***CFI* p.T62S**	3	0.63	0.89	0.26	(−3.56, 4.09)

Outer nasal RPE thickness

**Genotype**	Minimum observations	*P* value (univariable)	*P* value (multivariable)	Coefficient, µm (multivariable)	95% confidence interval, µm (multivariable)
***CFH* p.Y402H** **heterozygous vs WT**	15438	<0.001 ***	<0.001 ***	−0.24	(−0.33, −0.15)
***CFH* p.Y402H** **homozygous vs WT**	4757	<0.001 ***	<0.001 ***	−0.51	(−0.63, −0.38)
***CFH* p.Y402H** **homozygous vs heterozygous**	4757	<0.001 ***	NA	NA	NA
***ARMS2* p.A69S** **heterozygous vs WT**	11409	0.12	0.05	0.09	(0.00, 0.17)
***ARMS2* p.A69S** **homozygous vs WT**	1627	0.72	0.58	0.58	(−0.14, 0.25)
***ARMS2* p.A69S** **homozygous vs heterozygous**	1627	0.99	NA	NA	NA
***CFI* Type 1 RV**	85	0.009 **	0.002 **	−1.28	(−2.11, −0.45)
***CFI* VUS**	486	0.09	0.26	−0.20	(−0.54, 0.14)
***CFI* any RV**	582	0.01 *	0.04 *	−0.32	(−0.63, −0.01)
***CFI* p.G119R**	57	0.07	0.02 *	−1.21	(−2.19, −0.23)
***CFI* p.G287R**	14	0.05	0.04 *	−2.38	(−4.59, −0.17)
***CFI* p.A240G**	4	0.07	0.16	−2.51	(−6.00, 0.98)
***CFI* p.H418L**	3	0.38	0.10	4.15	(−0.78, 9.08)
***CFI* p.R474X**	2	0.27	0.21	−3.14	(−8.07, 1.79)
***CFI* p.P50A**	2	0.97	0.99	0.05	(−4.89, 4.98)
***CFI* p.I357M**	2	0.99	0.25	4.10	(−2.88, 11.08)
***CFI* p.G162D**	1	0.48	NA	NA	NA
***CFI* p.I340T**	8	0.29	0.20	1.60	(−0.87, 4.07)
***CFI* p.R406H**	193	0.88	0.57	0.16	(−0.39, 0.71)
***CFI* p.G261D**	190	0.04 *	0.02	−0.65	(−1.18, −0.12)
***CFI* p.K441R**	104	0.84	0.85	0.07	(−0.65, 0.79)
***CFI* p.T62S**	3	0.59	0.47	−3.14	(−8.07, 1.79)

Outer inferior RPE thickness

Genotype	Minimum observations	*P* value (univariable)	*P* value (multivariable)	Coefficient, µm (multivariable)	95% confidence interval, µm (multivariable)
***CFH* p.Y402H** **heterozygous vs WT**	15438	<0.001 ***	<0.001 ***	−0.29	(−0.35, −0.22)
***CFH* p.Y402H** **homozygous vs WT**	4757	<0.001 ***	<0.001 ***	−0.60	(−0.14, −0.01)
***CFH* p.Y402H** **homozygous vs heterozygous**	4757	<0.001 ***	NA	NA	NA
***ARMS2* p.A69S** **heterozygous vs WT**	11409	0.15	0.02 *	−0.08	(−0.14, −0.01)
***ARMS2* p.A69S** **homozygous vs WT**	1627	0.01 *	0.001 **	−0.24	(−0.39, −0.09)
***ARMS2* p.A69S** **homozygous vs heterozygous**	1627	0.13	NA	NA	NA
***CFI* Type 1 RV**	85	0.003 **	0.005 **	−0.88	(−1.50, −0.26)
***CFI* VUS**	486	0.006 **	0.17	−0.18	(−0.44, 0.08)
***CFI* any RV**	582	0.006 **	0.02 *	−0.28	(−0.51, −0.05)
***CFI* p.G119R**	57	0.08	0.04 *	−0.79	(−1.52, −0.05)
***CFI* p.G287R**	14	0.007 **	0.04 *	−1.77	(−3.42, −0.11)
***CFI* p.A240G**	4	0.009 **	0.02 *	−3.02	(−5.64, −0.41)
***CFI* p.H418L**	3	0.43	0.11	2.99	(−0.71, 6.69)
***CFI* p.R474X**	2	0.44	0.43	−1.49	(−5.19, 2.21)
***CFI* p.P50A**	2	0.74	0.73	0.66	(−3.04, 4.36)
***CFI* p.I357M**	2	0.95	0.32	2.66	(−2.57, 7.89)
***CFI* p.G162D**	1	0.97	NA	NA	NA
***CFI* p.I340T**	8	0.38	0.33	0.92	(−0.93, 2.77)
***CFI* p.R406H**	193	0.88	0.81	0.05	(−0.36, 0.47)
***CFI* p.G261D**	190	0.06	0.07	−0.37	(−0.76, 0.03)
***CFI* p.K441R**	104	0.14	0.46	−0.20	(−0.74, 0.34)
***CFI* p.T62S**	3	0.89	0.88	−0.28	(−3.97, 3.42)

Mean retinal thickness

**Genotype**	Minimum observations	*P* value (univariable)	*P* value (multivariable)	Coefficient, µm (multivariable)	95% confidence interval, µm (multivariable)
***CFH* p.Y402H** **heterozygous vs WT**	14420	0.06	0.007 **	−0.42	(−0.73, −0.12)
***CFH* p.Y402H** **homozygous vs WT**	4451	0.08	0.004 **	−0.64	(−1.08, −0.21)
***CFH* p.Y402H** **homozygous vs heterozygous**	4451	0.84	NA	NA	NA
***ARMS2* p.A69S** **heterozygous vs WT**	10579	0.58	0.75	0.05	(−0.25, 0.35)
***ARMS2* p.A69S** **homozygous vs WT**	1506	0.006 **	0.07	−0.62	(−1.28, 0.05)
***ARMS2* p.A69S** **homozygous vs heterozygous**	1506	0.03 *	NA	NA	NA
***CFI* Type 1 RV**	77	0.07	0.04 *	−3.01	(−5.88, −0.13)
***CFI* VUS**	452	0.26	0.99	0.01	(−1.18, 1.20)
***CFI* any RV**	539	0.09	0.55	−0.33	(−1.42, 0.76)
***CFI* p.G119R**	52	0.03 *	0.01 *	−4.26	(−0.17, −0.10)
***CFI* p.G287R**	12	0.81	0.77	−1.15	(−8.89, 6.60)
***CFI* p.A240G**	4	0.56	0.37	5.59	(−6.66, 17.84)
***CFI* p.H418L**	3	0.78	0.86	−1.57	(−18.89, 15.74)
***CFI* p.R474X**	2	0.47	0.50	4.90	(−9.23, 19.04)
***CFI* p.P50A**	1	0.12	NA	NA	NA
***CFI* p.I357M**	3	0.31	0.44	−9.60	(−34.10, 14.90)
***CFI* p.I340T**	8	0.92	0.99	0.09	(−8.57, 8.74)
***CFI* p.R406H**	176	0.26	0.29	1.50	(−0.89, 3.00)
***CFI* p.G261D**	178	0.82	0.89	0.13	(−1.72, 1.97)
***CFI* p.K441R**	99	0.20	0.27	−1.40	(−3.90, 1.10)
***CFI* p.T62S**	2	1.00	0.79	2.31	(−15.01, 19.62)

Central retinal thickness

**Genotype**	Minimum observations	*P* value (univariable)	*P* value (multivariable)	Coefficient, µm (multivariable)	95% confidence interval, µm (multivariable)
***CFH* p.Y402H** **heterozygous vs WT**	14420	0.99	0.24	−0.32	(−0.85, 0.21)
***CFH* p.Y402H** **homozygous vs WT**	4451	0.74	0.51	−0.25	(−1.01, 0.50)
***CFH* p.Y402H** **homozygous vs heterozygous**	4451	0.68	NA	NA	NA
***ARMS2* p.A69S** **heterozygous vs WT**	10579	<0.001 ***	0.002 **	−0.82	(−1.34, −0.29)
***ARMS2* p.A69S** **homozygous vs WT**	1506	<0.001 ***	<0.001 ***	−2.28	(−3.44, −1.13)
***ARMS2* p.A69S** **homozygous vs heterozygous**	1506	0.002 **	NA	NA	NA
***CFI* Type 1 RV**	77	0.44	0.13	−3.89	(−8.86, 1.08)
***CFI* VUS**	452	0.001 **	0.09	−1.79	(−3.85, 0.27)
***CFI* any RV**	539	0.001 **	0.04 *	−1.99	(−3.87, −0.11)
***CFI* p.G119R**	52	0.11	0.02 *	−7.17	(−13.05, −1.29)
***CFI* p.G287R**	12	0.46	0.36	6.29	(−7.11, 19.69)
***CFI* p.A240G**	4	1.00	0.80	2.70	(−18.50, 23.90)
***CFI* p.H418L**	3	0.85	0.63	−7.50	(−37.40, 25.20)
***CFI* p.R474X**	2	0.10	0.17	17.20	(−7.20, 41.70)
***CFI* p.P50A**	1	0.98	NA	NA	NA
***CFI* p.I357M**	3	0.66	0.69	−8.60	(−51.00, 33.80)
***CFI* p.I340T**	8	0.62	0.49	5.28	(−9.70, 20.26)
***CFI* p.R406H**	176	0.26	0.86	−0.30	(−3.66, 3.07)
***CFI* p.G261D**	178	0.27	0.91	0.18	(−3.01, 3.38)
***CFI* p.K441R**	99	0.006 **	0.001 **	−7.54	(−11.88, −3.21)
***CFI* p.T62S**	2	0.46	0.30	−15.70	(−45.70, 14.20)

Inner retinal thickness

**Genotype**	Minimum observations	*P* value (univariable)	*P* value (multivariable)	Coefficient, µm (multivariable)	95% confidence interval, µm (multivariable)
***CFH* p.Y402H** **heterozygous vs WT**	14420	0.04 *	0.002 **	−0.58	(−0.94, −0.22)
***CFH* p.Y402H** **homozygous vs WT**	4451	0.006 **	<0.001 ***	−1.11	(−1.62, −0.60)
***CFH* p.Y402H** **homozygous vs heterozygous**	4451	0.34	NA	NA	NA
***ARMS2* p.A69S** **heterozygous vs WT**	10579	0.05 *	0.24	−0.21	(−0.56, 0.14)
***ARMS2* p.A69S** **homozygous vs WT**	1506	<0.001 ***	0.005 **	−1.11	(−1.87, −0.34)
***ARMS2* p.A69S** **homozygous vs heterozygous**	1506	0.003 **	NA	NA	NA
***CFI* Type 1 RV**	77	0.29	0.12	−2.68	(−6.03, 0.67)
***CFI* VUS**	452	0.20	0.88	0.11	(−1.286, 1.50)
***CFI* any RV**	539	0.13	0.75	−0.21	(−1.48, 1.06)
***CFI* p.G119R**	52	0.14	0.04 *	−4.10	(−8.07, −0.13)
***CFI* p.G287R**	12	0.92	0.72	−1.63	(−10.68, 7.42)
***CFI* p.A240G**	4	0.49	0.23	8.84	(−5.47, 23.15)
***CFI* p.H418L**	3	0.97	0.79	−2.80	(−23.00, 17.40)
***CFI* p.R474X**	2	0.24	0.31	8.51	(−8.01, 25.03)
***CFI* p.P50A**	1	0.26	NA	NA	NA
***CFI* p.I357M**	3	0.45	0.56	−8.60	(−37.20, 20.10)
***CFI* p.I340T**	8	0.88	0.89	0.74	(−9.37, 10.86)
***CFI* p.R406H**	176	0.10	0.15	1.68	(−0.59, 3.95)
***CFI* p.G261D**	178	0.76	0.90	−0.14	(−2.30, 2.02)
***CFI* p.K441R**	99	0.35	0.29	−1.59	(−4.51, 1.34)
***CFI* p.T62S**	2	0.57	0.47	7.40	(−12.80, 27.60)

Outer retinal thickness

Genotype	Minimum observations	*P* value (univariable)	*P* value (multivariable)	Coefficient, µm (multivariable)	95% confidence interval, µm (multivariable)
***CFH* p.Y402H** **heterozygous vs WT**	14420	0.08	0.03 *	−0.35	(−0.67, −0.04)
***CFH* p.Y402H** **homozygous vs WT**	4451	0.16	0.04 *	−0.48	(−0.92, −0.03)
***CFH* p.Y402H** **homozygous vs heterozygous**	4451	0.95	NA	NA	NA
***ARMS2* p.A69S** **heterozygous vs WT**	10579	0.97	0.31	0.16	(−0.15, 0.47)
***ARMS2* p.A69S** **homozygous vs WT**	1506	0.07	0.22	−0.43	(−1.10, 0.25)
***ARMS2* p.A69S** **homozygous vs heterozygous**	1506	0.11	NA	NA	NA
***CFI* Type 1 RV**	77	0.05 *	0.04 *	−3.02	(−5.93, −0.11)
***CFI* VUS**	452	0.41	0.94	0.05	(−1.16, 1.26)
***CFI* any RV**	539	0.13	0.59	−0.31	(−1.41, 0.80)
***CFI* p.G119R**	52	0.03 *	0.02 *	−4.22	(−7.67, −0.77)
***CFI* p.G287R**	12	0.82	0.75	−1.29	(−9.14, 6.57)
***CFI* p.A240G**	4	0.63	0.47	4.73	(−7.70, 17.16)
***CFI* p.H418L**	3	0.74	0.91	−0.99	(−18.56, 16.58)
***CFI* p.R474X**	2	0.66	0.64	3.38	(−10.96, 17.73)
***CFI* p.P50A**	1	0.1	NA	NA	NA
***CFI* p.I357M**	3	0.29	0.44	−9.90	(−34.70, 15.00)
***CFI* p.I340T**	8	0.96	0.95	−0.30	(−9.09, 8.49)
***CFI* p.R406H**	176	0.47	0.36	0.92	(−1.06, 2.89)
***CFI* p.G261D**	178	0.90	0.83	0.20	(−1.67, 2.08)
***CFI* p.K441R**	99	0.23	0.39	−1.11	(−3.66, 1.43)
***CFI* p.T62S**	2	0.89	0.87	1.46	(−16.11, 19.03)

Temporal retinal thickness

Genotype	Minimum observations	*P* value (univariable)	*P* value (multivariable)	Coefficient, µm (multivariable)	95% confidence interval, µm (multivariable)
***CFH* p.Y402H** **heterozygous vs WT**	14420	0.11	0.01 *	−0.42	(−0.74, −0.10)
***CFH* p.Y402H** **homozygous vs WT**	4451	0.02 *	<0.001 ***	−0.87	(−1.33, −0.41)
***CFH* p.Y402H** **homozygous vs heterozygous**	4451	0.37	NA	NA	NA
***ARMS2* p.A69S** **heterozygous vs WT**	10579	0.06	0.32	−0.16	(−0.48, 0.16)
***ARMS2* p.A69S** **homozygous vs WT**	1506	<0.001 ***	0.004 **	−1.01	(−1.70, −0.32)
***ARMS2* p.A69S** **homozygous vs heterozygous**	1506	0.004 **	NA	NA	NA
***CFI* Type 1 RV**	77	0.21	0.13	−2.35	(−5.36, 0.66)
***CFI* VUS**	452	0.05	0.60	−0.33	(−1.58, 0.92)
***CFI* any RV**	539	0.02 *	0.37	−0.53	(−1.67, 0.62)
***CFI* p.G119R**	52	0.13	0.05 *	−3.62	(−7.18, −0.05)
***CFI* p.G287R**	12	0.96	0.72	−1.51	(−9.64, 6.62)
***CFI* p.A240G**	4	0.74	0.39	5.63	(−7.22, 18.49)
***CFI* p.H418L**	3	0.84	0.81	−2.24	(−20.42, 15.93)
***CFI* p.R474X**	2	0.48	0.59	4.10	(−10.74, 18.94)
***CFI* p.P50A**	1	0.43	NA	NA	NA
***CFI* p.I357M**	3	0.55	0.85	−2.50	(−28.20, 23.20)
***CFI* p.I340T**	8	0.90	1.00	0.03	(−9.06, 9.12)
***CFI* p.R406H**	176	0.02 *	0.70	0.41	(−1.64, 2.45)
***CFI* p.G261D**	178	0.69	0.87	0.16	(−1.78, 2.10)
***CFI* p.K441R**	99	0.17	0.15	−1.95	(−4.57, 0.68)
***CFI* p.T62S**	2	0.73	0.59	4.97	(−13.21, 23.14)

Superior retinal thickness

**Genotype**	Minimum observations	*P* value (univariable)	*P* value (multivariable)	Coefficient, µm (multivariable)	95% confidence interval, µm (multivariable)
***CFH* p.Y402H** **heterozygous vs WT**	14420	0.03 *	0.004 **	−0.50	(−0.84, −0.16)
***CFH* p.Y402H** **homozygous vs WT**	4451	0.01 *	<0.001 ***	−0.87	(−1.36, −0.39)
***CFH* p.Y402H** **homozygous vs heterozygous**	4451	0.53	NA	NA	NA
***ARMS2* p.A69S** **heterozygous vs WT**	10579	0.48	0.92	0.02	(−0.32, 0.35)
***ARMS2* p.A69S** **homozygous vs WT**	1506	0.001 **	0.02 *	−0.84	(−1.57, −0.11)
***ARMS2* p.A69S** **homozygous vs heterozygous**	1506	0.01 *	NA	NA	NA
***CFI* Type 1 RV**	77	0.04 *	0.02 *	−3.72	(−6.90, −0.53)
***CFI* VUS**	452	0.37	0.75	0.22	(−1.10, 1.54)
***CFI* any RV**	539	0.13	0.66	−0.27	(−1.48, 0.94)
***CFI* p.G119R**	52	0.02 *	0.01 *	−4.95	(−8.72, −1.17)
***CFI* p.G287R**	12	0.68	0.32	−4.38	(−12.98, 4.22)
***CFI* p.A240G**	4	0.55	0.35	6.51	(−7.09, 20.11)
***CFI* p.H418L**	3	0.94	0.79	−2.56	(−21.79, 16.67)
***CFI* p.R474X**	2	0.30	0.31	8.06	(−7.64, 23.76)
***CFI* p.P50A**	1	0.17	NA	NA	NA
***CFI* p.I357M**	3	0.37	0.47	−10.10	(−37.30, 17.10)
***CFI* p.I340T**	8	0.78	0.90	0.61	(−9.00, 10.23)
***CFI* p.R406H**	176	0.12	0.21	1.37	(−0.79, 3.53)
***CFI* p.G261D**	178	0.88	1.00	−0.01	(−2.06, 2.04)
***CFI* p.K441R**	99	0.48	0.54	−0.87	(−3.65, 1.91)
***CFI* p.T62S**	2	0.61	0.44	7.53	(−11.70, 26.75)

Nasal retinal thickness

**Genotype**	Minimum observations	*P* value (univariable)	*P* value (multivariable)	Coefficient, µm (multivariable)	95% confidence interval, µm (multivariable)
***CFH* p.Y402H** **heterozygous vs WT**	14420	0.04 *	0.006 **	−0.50	(−0.86, −0.15)
***CFH* p.Y402H** **homozygous vs WT**	4451	0.09	0.007 **	−0.69	(−1.20, −0.19)
***CFH* p.Y402H** **homozygous vs heterozygous**	4451	0.91	NA	NA	NA
***ARMS2* p.A69S** **heterozygous vs WT**	10579	0.54	0.99	−0.02	(−0.35, 0.35)
***ARMS2* p.A69S** **homozygous vs WT**	1506	0.006 **	0.07	−0.70	(−1.47, 0.07)
***ARMS2* p.A69S** **homozygous vs heterozygous**	1506	0.03 *	NA	NA	NA
***CFI* Type 1 RV**	77	0.12	0.05 *	−3.34	(−6.64, −0.03)
***CFI* VUS**	452	0.57	0.72	0.25	(−1.12, 1.63)
***CFI* any RV**	539	0.28	0.66	−0.27	(−1.48, 0.94)
***CFI* p.G119R**	52	0.06	0.03 *	−4.43	(−8.35, −0.51)
***CFI* p.G287R**	12	0.71	0.84	−0.91	(−9.84, 8.02)
***CFI* p.A240G**	4	0.52	0.31	7.34	(−6.78, 21.46)
***CFI* p.H418L**	3	0.56	0.55	−6.20	(−26.10, 13.80)
***CFI* p.R474X**	2	0.31	0.34	7.93	(−8.37, 24.24)
***CFI* p.P50A**	1	0.13	NA	NA	NA
***CFI* p.I357M**	3	0.15	0.17	−19.60	(−47.90, 8.60)
***CFI* p.I340T**	8	0.81	0.92	0.50	(−9.48, 10.48)
***CFI* p.R406H**	176	0.44	0.11	1.86	(−0.39, 4.10)
***CFI* p.G261D**	178	0.75	0.95	0.07	(−2.06, 2.20)
***CFI* p.K441R**	99	0.24	0.26	−1.64	(−4.53, 1.240)
***CFI* p.T62S**	2	0.85	0.70	4.00	(−16.00, 23.90)

Inferior retinal thickness

**Genotype**	Minimum observations	*P* value (univariable)	*P* value (multivariable)	Coefficient, µm (multivariable)	95% confidence interval, µm (multivariable)
***CFH* p.Y402H** heterozygous vs WT	14420	0.06	0.01 *	−0.43	(−0.76, −0.10)
***CFH* p.Y402H** homozygous vs WT	4451	0.05 *	0.003 **	−0.71	(−1.18, −0.25)
***CFH* p.Y402H** homozygous vs heterozygous	4451	0.71	NA	NA	NA
***ARMS2* p.A69S** heterozygous vs WT	10579	0.58	0.83	0.04	(−0.29, 0.36)
***ARMS2* p.A69S** homozygous vs WT	1506	0.01 *	0.10	−0.60	(−1.32, 0.11)
***ARMS2* p.A69S** homozygous vs heterozygous	1506	0.05	NA	NA	NA
***CFI* Type 1 RV**	77	0.26	0.19	−2.07	(−5.14, 0.99)
***CFI* VUS**	452	0.34	0.80	0.17	(−1.10, 1.44)
***CFI* any RV**	539	0.18	0.93	−0.05	(−1.22, 1.11)
***CFI* p.G119R**	52	0.10	0.06	−3.54	(−7.17, 0.10)
***CFI* p.G287R**	12	0.51	0.82	0.97	(−7.31, 9.24)
***CFI* p.A240G**	4	0.43	0.25	7.66	(−5.42, 20.74)
***CFI* p.H418L**	3	0.86	0.72	3.36	(−15.14, 21.86)
***CFI* p.R474X**	2	0.58	0.63	3.69	(−11.41, 18.80)
***CFI* p.P50A**	1	0.08	NA	NA	NA
***CFI* p.I357M**	3	0.54	0.72	−4.70	(−30.90, 21.50)
***CFI* p.I340T**	8	0.99	0.96	−0.26	(−9.51, 8.99)
***CFI* p.R406H**	176	0.66	0.14	1.56	(−0.52, 3.64)
***CFI* p.G261D**	178	0.99	0.92	−0.10	(−2.08, 1.87)
***CFI* p.K441R**	99	0.32	0.49	−0.94	(−3.61, 1.74)
***CFI* p.T62S**	2	0.93	0.90	1.25	(−17.25, 19.75)

Inner temporal retinal thickness

**Genotype**	Minimum observations	*P* value (univariable)	*P* value (multivariable)	Coefficient, µm (multivariable)	95% confidence interval, µm (multivariable)
***CFH* p.Y402H** heterozygous vs WT	14420	0.06	0.002 **	−0.58	(−0.94, −0.21)
***CFH* p.Y402H** homozygous vs WT	4451	0.004 **	<0.001 ***	−1.18	(−1.70, −0.66)
***CFH* p.Y402H** homozygous vs heterozygous	4451	0.23	NA	NA	NA
***ARMS2* p.A69S** heterozygous vs WT	10579	0.003 **	0.03 *	−0.41	(−0.77, −0.05)
***ARMS2* p.A69S** homozygous vs WT	1506	<0.001 ***	<0.001 ***	−1.46	(−2.26, −0.67)
***ARMS2* p.A69S** homozygous vs heterozygous	1506	0.002 **	NA	NA	NA
***CFI* Type 1 RV**	77	0.60	0.32	−1.73	(−5.14, 1.68)
***CFI* VUS**	452	0.08	0.84	−0.15	(−1.56, 1.27)
***CFI* any RV**	539	0.07	0.67	−0.29	(−1.58, 1.01)
***CFI* p.G119R**	52	0.25	0.08	−3.59	(−7.63, 0.45)
***CFI* p.G287R**	12	0.80	0.94	0.35	(−8.86, 9.55)
***CFI* p.A240G**	4	0.49	0.22	9.19	(−5.37, 23.74)
***CFI* p.H418L**	3	0.84	0.98	−0.30	(−20.90, 20.30)
***CFI* p.R474X**	2	0.26	0.36	7.92	(−8.88, 24.73)
***CFI* p.P50A**	1	0.64	NA	NA	NA
***CFI* p.I357M**	3	0.77	0.93	−1.30	(−30.40, 27.80)
***CFI* p.I340T**	8	0.99	0.96	0.27	(−10.02, 10.56)
***CFI* p.R406H**	176	0.02 *	0.32	1.19	(−1.13, 3.50)
***CFI* p.G261D**	178	0.54	0.88	0.17	(−2.03, 2.36)
***CFI* p.K441R**	99	0.19	0.11	−2.40	(−5.37, 0.58)
***CFI* p.T62S**	2	0.76	0.72	3.80	(−16.80, 24.40)

Inner superior retinal thickness

**Genotype**	Minimum observations	*P* value (univariable)	*P* value (multivariable)	Coefficient, µm (multivariable)	95% confidence interval, µm (multivariable)
CFH **p.Y402H heterozygous vs WT**	14420	0.02 *	<0.001 ***	−0.62	(−1.01, −0.24)
CFH **p.Y402H homozygous vs WT**	4451	0.004 **	<0.001 ***	−1.12	(−1.66, −0.57)
CFH **p.Y402H homozygous vs heterozygous**	4451	0.41	NA	NA	NA
ARMS2 **p.A69S heterozygous vs WT**	10579	0.26	0.67	−0.08	(−0.46, 0.29)
ARMS2 **p.A69S homozygous vs WT**	1506	<0.001 ***	0.02 *	−1.02	(−1.84, −0.20)
ARMS2 **p.A69S homozygous vs heterozygous**	1506	0.010 **	NA	NA	NA
CFI **Type 1 RV**	77	0.14	0.05	−3.53	(−7.10, 0.05)
CFI **VUS**	452	0.47	0.76	0.26	(−1.23, 1.74)
CFI **any RV**	539	0.24	0.76	−0.21	(−1.57, 1.14)
CFI **p.G119R**	52	0.08	0.03 *	−4.69	(−8.92, −0.45)
CFI **p.G287R**	12	0.87	0.42	−3.99	(−13.63, 5.66)
CFI **p.A240G**	4	0.51	0.27	8.62	(−6.64, 23.88)
CFI **p.H418L**	3	0.94	0.69	−4.40	(−26.00, 17.10)
CFI **p.R474X**	2	0.26	0.30	9.26	(−8.36, 26.87)
CFI **p.P50A**	1	0.19	NA	NA	NA
CFI **p.I357M**	3	0.30	0.36	−14.2	(−44.80, 16.30)
CFI **p.I340T**	8	0.85	0.92	0.56	(−10.22, 11.35)
CFI **p.R406H**	176	0.12	0.25	1.41	(−1.01, 3.84)
CFI **p.G261D**	178	0.67	0.90	0.15	(−2.15, 2.45)
CFI **p.K441R**	99	0.55	0.50	−1.08	(−4.20, 2.04)
CFI **p.T62S**	2	0.52	0.40	9.20	(−12.40, 30.80)

Inner nasal retinal thickness

**Genotype**	Minimum observations	*P* value (univariable)	*P* value (multivariable)	Coefficient, µm (multivariable)	95% confidence interval, µm (multivariable)
***CFH* p.Y402H** **heterozygous vs WT**	14420	0.04 *	0.002 **	−0.60	(−0.98, −0.22)
***CFH* p.Y402H** **homozygous vs WT**	4451	0.02 *	<0.001 ***	−1.06	(−1.61, −0.52)
***CFH* p.Y402H** **homozygous vs heterozygous**	4451	0.54	NA	NA	NA
***ARMS2* p.A69S** **heterozygous vs WT**	10579	0.05	0.23	0.23	(−0.60, 0.15)
***ARMS2* p.A69S** **homozygous vs WT**	1506	<0.001 ***	0.006 **	−1.16	(−1.98, −0.34)
***ARMS2* p.A69S** **homozygous vs heterozygous**	1506	0.005 **	NA	NA	NA
***CFI* Type 1 RV**	77	0.36	0.15	−2.65	(−6.23, 0.93)
***CFI* VUS**	452	0.24	0.89	0.10	(−1.38, 1.59)
***CFI* any RV**	539	0.18	0.80	−0.18	(−1.53, 1.18)
***CFI* p.G119R**	52	0.27	0.10	−3.57	(−7.81, 0.68)
***CFI* p.G287R**	12	0.89	0.71	−1.83	(−11.50, 7.84)
***CFI* p.A240G**	4	0.64	0.33	7.58	(−7.71, 22.88)
***CFI* p.H418L**	3	0.69	0.58	−6.10	(−27.70, 15.50)
***CFI* p.R474X**	2	0.24	0.30	9.31	(−8.34, 26.97)
***CFI* p.P50A**	1	0.26	NA	NA	NA
***CFI* p.I357M**	3	0.24	0.29	−16.40	(−47.00, 14.20)
***CFI* p.I340T**	8	0.71	0.72	2.00	(−8.81, 12.81)
***CFI* p.R406H**	176	0.13	0.13	1.86	(−0.57, 4.29)
***CFI* p.G261D**	178	0.78	0.85	−0.22	(−2.52, 2.09)
***CFI* p.K441R**	99	0.32	0.26	−1.81	(−4.94, 1.32)
***CFI* p.T62S**	2	0.52	0.44	8.60	(−13.10, 30.20)

Inner inferior retinal thickness

**Genotype**	Minimum observations	*P* value (univariable)	*P* value (multivariable)	Coefficient, µm (multivariable)	95% confidence interval, µm (multivariable)
***CFH* p.Y402H** **heterozygous vs WT**	14420	0.17	0.017 *	−0.47	(−0.85, −0.09)
***CFH* p.Y402H** **homozygous vs WT**	4451	0.02 *	<0.001 ***	−1.03	(−1.57, −0.48)
***CFH* p.Y402H** **homozygous vs heterozygous**	4451	0.36	NA	NA	NA
***ARMS2* p.A69S** **heterozygous vs WT**	10579	0.18	0.53	−0.12	(−0.50, 0.26)
***ARMS2* p.A69S** **homozygous vs WT**	1506	<0.001 ***	0.01 *	−1.03	(−1.85, −0.21)
***ARMS2* p.A69S** **homozygous vs heterozygous**	1506	0.005 **	NA	NA	NA
***CFI* Type 1 RV**	77	0.27	0.12	−2.82	(−6.40, 0.76)
***CFI* VUS**	452	0.21	0.78	0.21	(−1.28, 1.69)
***CFI* any RV**	539	0.13	0.82	−0.16	(−1.51, 1.20)
***CFI* p.G119R**	52	0.09	0.04 *	−4.51	(−8.75, −0.27)
***CFI* p.G287R**	12	0.85	0.83	−1.05	(−10.71, 8.60)
***CFI* p.A240G**	4	0.92	0.20	9.98	(−5.29, 25.25)
***CFI* p.H418L**	3	0.54	0.97	−0.40	(−22.00, 21.20)
***CFI* p.R474X**	2	0.91	0.40	7.55	(−10.08, 25.17)
***CFI* p.P50A**	1	0.31	NA	NA	NA
***CFI* p.I357M**	3	0.41	0.88	−2.30	(−32.80, 28.30)
***CFI* p.I340T**	8	0.82	0.98	0.14	(−10.65, 10.94)
***CFI* p.R406H**	176	0.04 *	0.07	2.26	(−0.17, 4.68)
***CFI* p.G261D**	178	0.91	0.57	−0.67	(−2.97, 1.63)
***CFI* p.K441R**	99	0.22	0.51	−1.05	(−4.18, 2.07)
***CFI* p.T62S**	2	0.74	0.47	8.00	(−13.60, 29.60)

Outer temporal retinal thickness

**Genotype**	Minimum observations	*P* value (univariable)	*P* value (multivariable)	Coefficient, µm (multivariable)	95% confidence interval, µm (multivariable)
***CFH* p.Y402H** **heterozygous vs WT**	14420	0.36	0.16	−0.24	(−0.58, 0.10)
***CFH* p.Y402H** **homozygous vs WT**	4451	0.19	0.03 *	−0.54	(−1.02, −0.07)
***CFH* p.Y402H** **homozygous vs heterozygous**	4451	0.70	NA	NA	NA
***ARMS2* p.A69S** **heterozygous vs WT**	10579	0.67	0.61	0.09	(−0.25, 0.42)
***ARMS2* p.A69S** **homozygous vs WT**	1506	0.01 *	0.04 *	−0.75	(−1.47, −0.03)
***ARMS2* p.A69S** **homozygous vs heterozygous**	1506	0.05 *	NA	NA	NA
***CFI* Type 1 RV**	77	0.07	0.06	−2.97	(−6.11, 0.17)
***CFI* VUS**	452	0.07	0.44	−0.52	(−1.82, 0.79)
***CFI* any RV**	539	0.02 *	0.21	−0.77	(−1.96, 0.42)
***CFI* p.G119R**	52	0.01 *	0.06	−3.58	(−7.30, 0.14)
***CFI* p.G287R**	12	0.52	0.44	−3.37	(−11.85, 5.11)
***CFI* p.A240G**	4	0.65	0.76	2.08	(−11.33, 15.49)
***CFI* p.H418L**	3	0.94	0.67	−4.18	(−23.14, 14.78)
***CFI* p.R474X**	2	0.41	0.97	0.28	(−15.20, 15.76)
***CFI* p.P50A**	1	0.20	NA	NA	NA
***CFI* p.I357M**	3	0.53	0.78	−3.80	(−30.60, 23.10)
***CFI* p.I340T**	8	0.72	0.97	−0.21	(−9.69, 9.27)
***CFI* p.R406H**	176	0.17	0.73	−0.37	(−2.50, 1.76)
***CFI* p.G261D**	178	0.87	0.88	0.16	(−1.87, 2.18)
***CFI* p.K441R**	99	0.45	0.29	−1.49	(−4.24, 1.25)
***CFI* p.T62S**	2	0.76	0.53	6.11	(−12.84, 25.07)

Outer superior retinal thickness

**Genotype**	Minimum observations	*P* value (univariable)	*P* value (multivariable)	Coefficient, µm (multivariable)	95% confidence interval, µm (multivariable)
***CFH* p.Y402H** **heterozygous vs WT**	14420	0.13	0.03 *	−0.37	(−0.71, −0.03)
***CFH* p.Y402H** **homozygous vs WT**	4451	0.10	0.009 **	−0.64	(−1.12, −0.16)
***CFH* p.Y402H** **homozygous vs heterozygous**	4451	0.75	NA	NA	NA
***ARMS2* p.A69S** **heterozygous vs WT**	10579	0.83	0.49	0.12	(−0.22, 0.45)
***ARMS2* p.A69S** **homozygous vs WT**	1506	0.01 *	0.10	−0.62	(−1.35, 0.12)
***ARMS2* p.A69S** **homozygous vs heterozygous**	1506	0.03*	NA	NA	NA
***CFI* Type 1 RV**	77	0.02 *	0.02 *	−3.93	(−7.09, −0.77)
***CFI* VUS**	452	0.33	0.79	0.18	(−1.13, 1.49)
***CFI* any RV**	539	0.08	0.60	−0.32	(−1.52, 0.87)
***CFI* p.G119R**	52	0.02 *	0.007 **	−5.19	(−8.93, −1.44)
***CFI* p.G287R**	12	0.57	0.27	−4.77	(−13.30, 3.76)
***CFI* p.A240G**	4	0.47	0.52	4.41	(−9.08, 17.89)
***CFI* p.H418L**	3	0.49	0.94	−0.68	(−19.75, 18.39)
***CFI* p.R474X**	2	0.49	0.39	6.86	(−8.71, 22.43)
***CFI* p.P50A**	1	0.08	NA	NA	NA
***CFI* p.I357M**	3	0.13	0.67	−5.9	(−32.90, 21.10)
***CFI* p.I340T**	8	0.95	0.89	0.66	(−8.87, 10.20)
***CFI* p.R406H**	176	0.48	0.23	1.33	(−0.82, 3.47)
***CFI* p.G261D**	178	0.76	0.88	−0.16	(−2.20, 1.87)
***CFI* p.K441R**	99	0.23	0.64	−0.66	(−3.42, 2.10)
***CFI* p.T62S**	2	0.77	0.55	5.85	(−13.22, 24.91)

Outer nasal retinal thickness

**Genotype**	Minimum observations	*P* value (univariable)	*P* value (multivariable)	Coefficient, µm (multivariable)	95% confidence interval, µm (multivariable)
***CFH* p.Y402H** **heterozygous vs WT**	14420	0.10	0.04 *	−0.40	(−0.78, −0.02)
***CFH* p.Y402H** **homozygous vs WT**	4451	0.49	0.25	−0.32	(−0.85, 0.22)
***CFH* p.Y402H** **homozygous vs heterozygous**	4451	0.94	NA	NA	NA
***ARMS2* p.A69S** **heterozygous vs WT**	10579	0.91	0.21	0.24	(−0.13, 0.61)
***ARMS2* p.A69S** **homozygous vs WT**	1506	0.34	0.59	−0.23	(−1.05, 0.59)
***ARMS2* p.A69S** **homozygous vs heterozygous**	1506	0.27	NA	NA	NA
***CFI* Type 1 RV**	77	0.05 *	0.03 *	−3.95	(−7.47, −0.43)
***CFI* VUS**	452	0.87	0.59	0.41	(−1.06, 1.87)
***CFI* any RV**	539	0.53	0.79	−0.18	(−1.53, 1.15)
***CFI* p.G119R**	52	0.11	0.01 *	−5.34	(−9.51, −1.17)
***CFI* p.G287R**	12	0.87	1.00	0.01	(−9.49, 9.51)
***CFI* p.A240G**	4	0.43	0.36	7.09	(−7.93, 22.11)
***CFI* p.H418L**	3	0.91	0.57	−6.20	(−27.40, 15.00)
***CFI* p.R474X**	2	0.31	0.46	6.55	(−10.79, 23.89)
***CFI* p.P50A**	1	0.17	NA	NA	NA
***CFI* p.I357M**	3	0.74	0.14	−22.80	(−52.80, 7.20)
***CFI* p.I340T**	8	0.97	0.85	−1.00	(−11.62, 9.62)
***CFI* p.R406H**	176	0.32	0.13	1.85	(−0.54, 4.24)
***CFI* p.G261D**	178	0.88	0.76	0.35	(−1.91, 2.62)
***CFI* p.K441R**	99	0.48	0.35	−1.48	(−4.55, 1.59)
***CFI* p.T62S**	2	0.56	0.96	−0.60	(−21.80, 20.60)

Outer inferior retinal thickness

**Genotype**	Minimum observations	*P* value (univariable)	*P* value (multivariable)	Coefficient, µm (multivariable)	95% confidence interval, µm (multivariable)
***CFH* p.Y402H** **heterozygous vs WT**	14420	0.07	0.03 *	−0.40	(−0.76, −0.05)
***CFH* p.Y402H** **homozygous vs WT**	4451	0.26	0.11	−0.41	(−0.92, 0.10)
***CFH* p.Y402H** **homozygous vs heterozygous**	4451	1.00	NA	NA	NA
***ARMS2* p.A69S** **heterozygous vs WT**	10579	1.00	0.29	0.19	(−0.16, 0.54)
***ARMS2* p.A69S** **homozygous vs WT**	1506	0.66	0.73	−0.14	(−0.92, 0.64)
***ARMS2* p.A69S** **homozygous vs heterozygous**	1506	0.66	NA	NA	NA
***CFI* Type 1 RV**	77	0.36	0.44	−1.33	(−4.67, 2.02)
***CFI* VUS**	452	0.67	0.86	0.13	(−1.26, 1.51)
***CFI* any RV**	539	0.43	0.94	0.05	(−1.22, 1.32)
***CFI* p.G119R**	52	0.18	0.21	−2.52	(−6.48, 1.44)
***CFI* p.G287R**	12	0.31	0.52	2.99	(−6.03, 12.01)
***CFI* p.A240G**	4	0.55	0.46	5.34	(−8.92, 19.61)
***CFI* p.H418L**	3	0.84	0.49	7.10	(−13.10, 27.30)
***CFI* p.R474X**	2	0.94	0.98	−0.16	(−16.64, 16.31)
***CFI* p.P50A**	1	0.08	NA	NA	NA
***CFI* p.I357M**	3	0.44	0.62	−7.10	(−35.70, 21.40)
***CFI* p.I340T**	8	0.99	0.90	−0.66	(−10.75, 9.43)
***CFI* p.R406H**	176	1.00	0.45	0.87	(−1.40, 3.13)
***CFI* p.G261D**	178	0.85	0.67	0.46	(−1.69, 2.61)
***CFI* p.K441R**	99	0.29	0.58	−0.82	(−3.74, 2.09)
***CFI* p.T62S**	2	0.42	0.59	−5.50	(−25.70, 14.70)
