# Supplementary material for: Rare complement factor I variants associated with reduced macular thickness and age-related macular degeneration in the UK Biobank
Source: Hum Mol Genet. 2022 Mar 14;31(16):2678–92. doi: 10.1093/hmg/ddac060 (PMC9402241; doi:10.1093/hmg/ddac060)
Supplement: Supplemental_Appendix_2_ddac060 [file supplemental_appendix_2_ddac060.pdf]

Supplemental Appendix 2 – UK Biobank Eye and Vision Consortium Member List

The most recent version is available at: ukbiobankeyeconsortium.org.uk/people

Steering Committee

Chair: Prof Andrew LOTERY, Southampton

Co-Chair: Prof Andrew DICK, Bristol

Prof Paul BISHOP, Manchester

Prof Simon HARDING, Liverpool

Prof Bal DHILLON, Edinburgh

Prof Sir Peng Tee KHAW, London

Prof James MORGAN, Cardiff

Consortium Members

Prof Naomi ALLEN, University of Oxford

Prof Tariq ASLAM, Manchester University

Dr Denize ATAN, University of Bristol

Prof Sarah BARMAN, Kingston University

Prof Jenny BARRETT, University of Leeds

Prof Paul BISHOP, Manchester University

Prof Graeme BLACK, The University of Manchester

Dr Catey BUNCE, King's College London

Dr Roxana CARARE, University of Southampton

Prof Usha CHAKRAVARTHY, Queens University, Belfast

Miss Michelle CHAN, Moorfields Eye Hospital, London

Dr Sharon CHUA, UCL Institute of Ophthalmology

Dr Valentina CIPRIANI, UCL Institute of Ophthalmology

Dr Alexander DAY, Moorfields Eye Hospital, London

Miss Parul DESAI, Moorfields Eye Hospital, London

Prof Bal DHILLON, University of Edinburgh

Prof Andrew DICK, University of Bristol

Dr Alexander DONEY, University of Dundee

Dr Cathy EGAN, Moorfields Eye Hospital, London

Prof Sarah ENNIS, University of Southampton

Prof Paul FOSTER, UCL Institute of Ophthalmology

Dr Marcus FRUTTIGER, UCL Institute of Ophthalmology

Dr John GALLACHER, University of Oxford

Prof David (Ted) GARWAY-HEATH, UCL Institute of Ophthalmology

Dr Jane GIBSON, University of Southampton

Mr Dan GORE, Moorfields Eye Hospital, London

Prof Jeremy GUGGENHEIM, Cardiff University

Prof Chris HAMMOND, King's College London

Prof Alison HARDCASTLE, UCL Institute of Ophthalmology

Prof Simon HARDING, University of Liverpool

Dr Ruth HOGG, Queen's University, Belfast

Dr Pirro HYSI, King's College London

Mr Pearse A KEANE, UCL Institute of Ophthalmology

Prof Sir Peng Tee KHAW, UCL Institute of Ophthalmology

Dr Anthony KHAWAJA, Moorfields Eye Hospital, London

Mr Gerassimos LASCARATOS, Moorfields Eye Hospital, London

Dr Thomas LITTLEJOHNS, University of Oxford

Prof Andrew LOTERY- University of Southampton

Prof Phil LUTHERT, UCL Institute of Ophthalmology

Dr Tom MACGILLIVRAY, University of Edinburgh

Dr Sarah MACKIE, University of Leeds

Dr Bernadette MCGUINNESS, Queen’s University Belfast

Dr Gareth MCKAY, Queen's University Belfast

Mr Martin MCKIBBIN, Leeds Teaching Hospitals NHS Trust

Dr Danny MITRY, University of Edinburgh

Prof Tony MOORE, UCL Institute of Ophthalmology

Prof James MORGAN, Cardiff University

Ms Zaynah MUTHY, UCL Institute of Ophthalmology

Mr Eoin O'SULLIVAN, King's College Hospital

Dr Chris OWEN, St George's, University of London

Mr Praveen PATEL, Moorfields Eye Hospital, London

Mr Euan PATERSON, Queens University Belfast

Dr Tunde PETO, Queen's University Belfast

Dr Axel PETZOLD, UCL Institute of Neurology

Dr Nikolas PONTIKOS, UCL Institute of Ophthalmology

Prof Jugnoo RAHI, UCL Institute of Child Health

Dr Alicja RUDNICKA, St George's, University of London

Mr Jay SELF, University of Southampton

Prof Sobha SIVAPRASAD, Moorfields Eye Hospital, London

Mr David STEEL, Newcastle University

Mrs Irene STRATTON, Gloucestershire Hospitals NHS Foundation Trust

Mr Nicholas STROUTHIDIS, Moorfields Eye Hospital, London

Prof Cathie SUDLOW, University of Edinburgh

Dr Robyn TAPP, St George's, University of London

Dr Caroline THAUNG, UCL Institute of Ophthalmology

Miss Dhanes THOMAS, Moorfields Eye Hospital, London

Prof Emanuele TRUCCO, University of Dundee

Mr Adnan TUFAIL, Moorfields Eye Hospital, London

Dr Veronique VITART, University of Edinburgh

Prof Stephen VERNON, University Hospital, Nottingham

Mr Ananth VISWANATHAN, Moorfields Eye Hospital, London

Miss Cathy WILLIAMS, University of Bristol

Dr Katie WILLIAMS, King's College London

Prof Jayne WOODSIDE, Queen's University Belfast

Dr Max YATES, University of East Anglia

Ms Jennifer YIP, University of Cambridge

Dr Yalin ZHENG, University of Liverpool
