# Supplementary material for: Rare complement factor I variants associated with reduced macular thickness and age-related macular degeneration in the UK Biobank
Source: Hum Mol Genet. 2022 Mar 14;31(16):2678–92. doi: 10.1093/hmg/ddac060 (PMC9402241; doi:10.1093/hmg/ddac060)
Supplement: Supplemental_Figure_1_ddac060 [file supplemental_figure_1_ddac060.pdf]

Supplemental Figure 1. Visual check of multivariable linear model assumptions for mean RPE-BM (A) and retinal (B) thicknesses at the macula including all covariates selected for stepwise linear regression. Univariable linear model assumptions between *CFI* type 1 RVs and mean RPE-BM (C) and retinal (D) thicknesses are also shown.

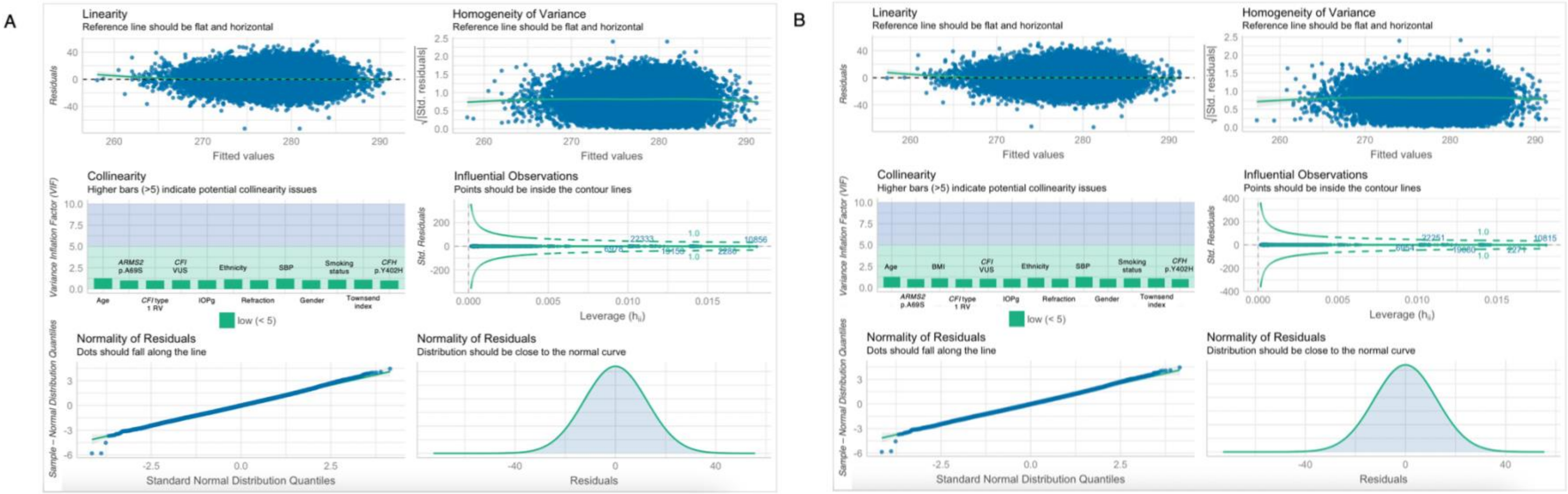

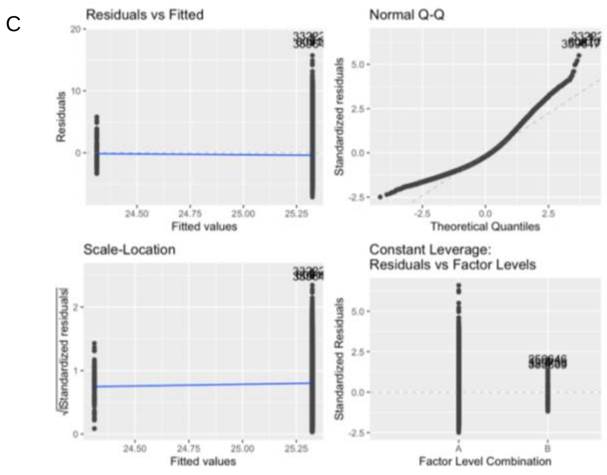

H

mass

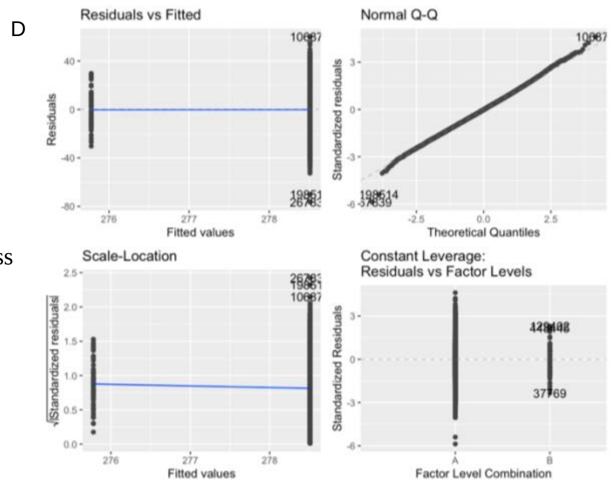

Abbreviations: *ARMS2* = age-related maculopathy susceptibility protein 2 gene, *CFH* = complement factor gene, *CFI* = complement factor I gene, BMI = body index, IOPg = goldmann-corrected intraocular pressure, RPE-BM = retinal pigment epithelium-Bruch's membrane complex, RV = rare variant, SBP = systolic blood pressure, VUS = variant of uncertain significance.
