# Supplementary material for: Rare complement factor I variants associated with reduced macular thickness and age-related macular degeneration in the UK Biobank
Source: Hum Mol Genet. 2022 Mar 14;31(16):2678–92. doi: 10.1093/hmg/ddac060 (PMC9402241; doi:10.1093/hmg/ddac060)

**Supplemental Figure 2.** Prevalence of *CFI* type 1 rare variant carrier status at each year of age in participants who underwent optical coherence tomography imaging as well as in the overall UK Biobank cohort. Linear model regression lines (black for Imaged and gray for Overall) and 95% confidence intervals (light gray bands) are shown. Fitted coefficients, $R^2$, and *P* values are indicated above each plot.

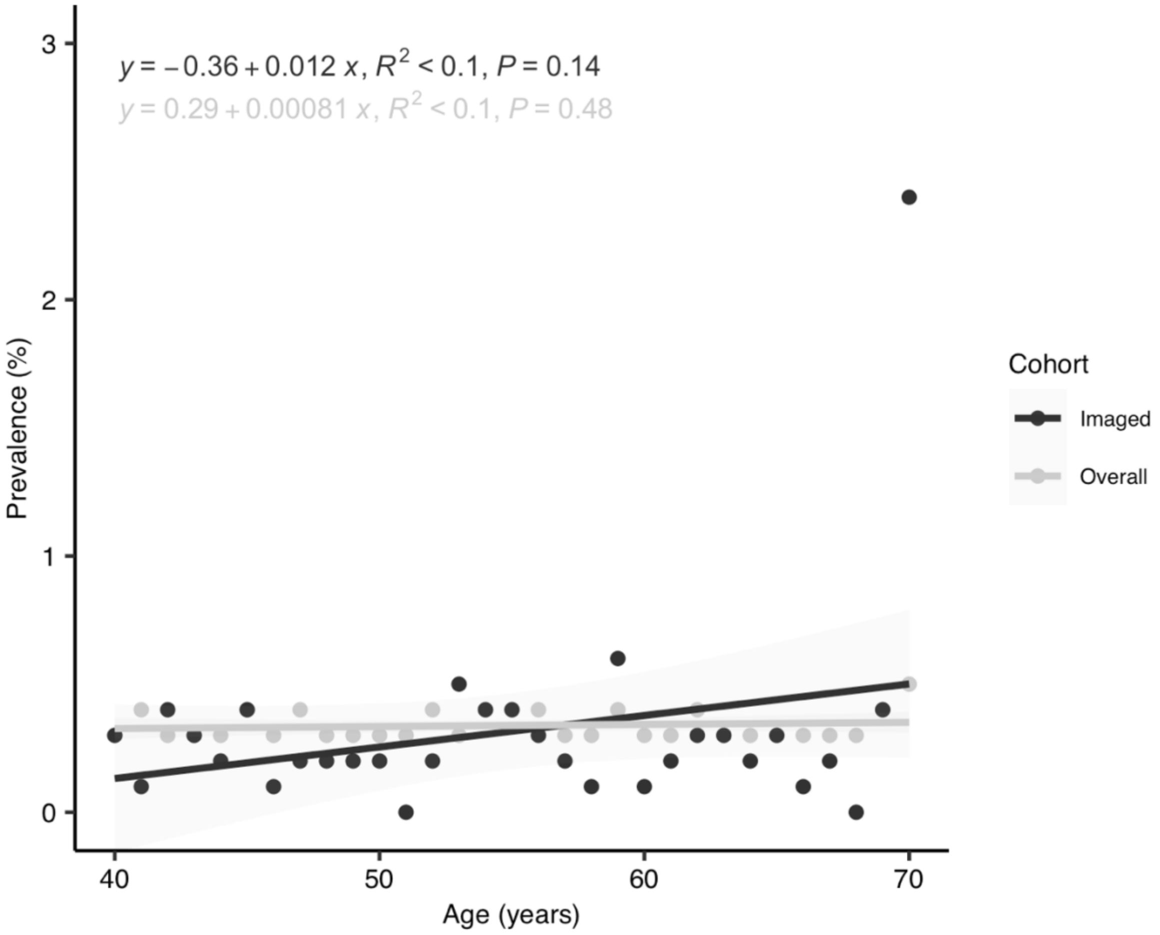

Supplement: Supplemental_Figure_2_ddac060 [file supplemental_figure_2_ddac060.pdf]
