# Supplementary material for: Rare complement factor I variants associated with reduced macular thickness and age-related macular degeneration in the UK Biobank
Source: Hum Mol Genet. 2022 Mar 14;31(16):2678–92. doi: 10.1093/hmg/ddac060 (PMC9402241; doi:10.1093/hmg/ddac060)
Supplement: Supplemental_Figure_3_ddac060 [file supplemental_figure_3_ddac060.pdf]

Supplemental Figure 3. Distribution of macular RPE-BM (A) and retinal (B) thicknesses at different ETDRS macular subfields between *CFI* type 1 RVs (blue) and VUS (yellow) carriers. *P* values for hypothesis testing were computed using an F-test, with significant values indicated by * for $P<0.05$. Individuals with a health record diagnosis of age-related macular degeneration or missing *CFI* genotype data were excluded.

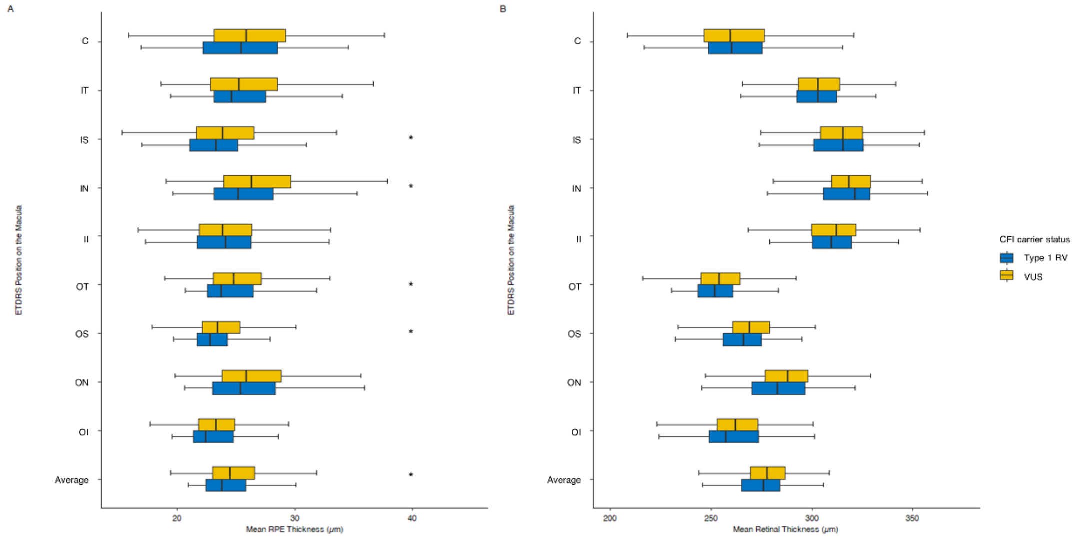

Abbrevations: C = central macular subfield, *CFI* = complement factor I gene, ETDRS = Early Treatment Diabetic Retinopathy Study, II = inner inferior subfield, IN = inner nasal subfield, IS = inner superior subfield, IT = inner temporal subfield, OI = outer inferior subfield, ON = outer nasal subfield, OS = outer superior subfield, OT = outer temporal subfield, RPE-BM = retinal pigment epithelium-Bruch's membrane complex, RV = rare variant, VUS = variant of uncertain significance.
