# Supplementary material for: Rare complement factor I variants associated with reduced macular thickness and age-related macular degeneration in the UK Biobank
Source: Hum Mol Genet. 2022 Mar 14;31(16):2678–92. doi: 10.1093/hmg/ddac060 (PMC9402241; doi:10.1093/hmg/ddac060)
Supplement: Supplemental_Figure_4_ddac060 [file supplemental_figure_4_ddac060.pdf]

Supplemental Figure 4. Cross-sectional mean RPE-BM (A) and retinal (B) thicknesses at each year of age plotted for *CFI* type 1 RVs and VUS carriers. Each point represents the mean macular thickness of the relevant group at that year of age. Individuals with a health record diagnosis of age-related macular degeneration or missing *CFI* genotype data were excluded. Linear model regression lines (blue for *CFI* type 1 RVs and yellow for *CFI* VUS) and 95% confidence intervals (light gray bands) are shown. Fitted coefficients, R^2, and P values are indicated above each plot. There was no significant difference in the slopes of regression lines (P=0.50 for RPE and P=0.08 for retina).

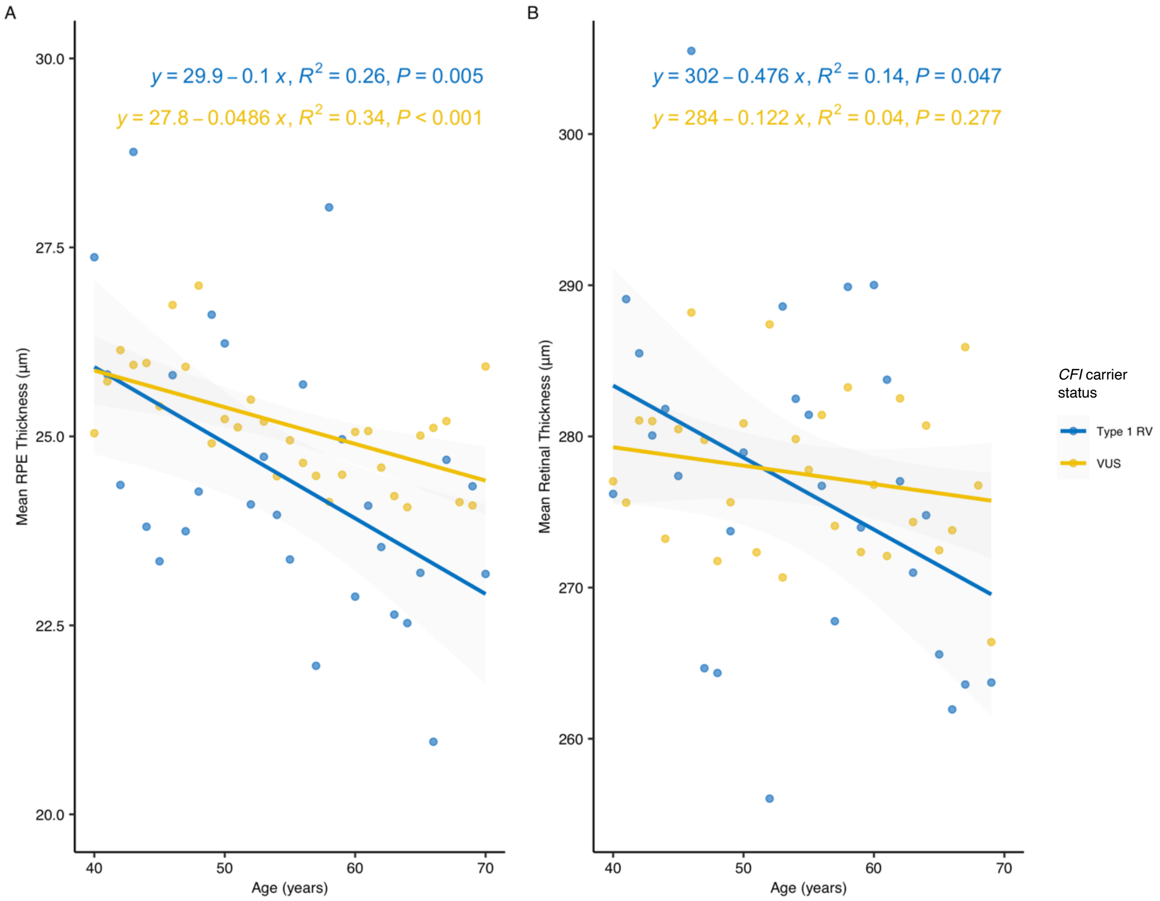

Abbrevations: *CFI* = complement factor I gene, RPE-BM = retinal pigment epithelium-Bruch's membrane complex, RV = rare variant, VUS = variant of uncertain significance.
