# Supplementary material for: Rare complement factor I variants associated with reduced macular thickness and age-related macular degeneration in the UK Biobank
Source: Hum Mol Genet. 2022 Mar 14;31(16):2678–92. doi: 10.1093/hmg/ddac060 (PMC9402241; doi:10.1093/hmg/ddac060)
Supplement: Supplemental_Table_1_ddac060 [file supplemental_table_1_ddac060.pdf]

Supplemental Table 1. Overview of *CFI* variant compound heterozygous participants and their OCT-derived macular imaging status and health-derived diagnosis of AMD.

**Genotype 1**	**Genotype 2**	***N***	***N* imaged**	***N* diagnosis of AMD**
CFI type 1 and type 1 RVs (*n* = 2)				
p.A240G	p.P50A	1	0	0
p.I357M	p.R474X	1	0	0
CFI type 1 and type 2 RVs (*n* = 1)				
p.A431T	p.I340T	1	0	0
CFI type 1 RV and VUS (*n* = 13)				
p.G119R	p.G261D	1	0	0
p.I357M	p.G261D	1	0	0
p.H418L	p.G261D	1	0	0
p.A431T	p.G261D	1	0	0
p.R474X	p.G261D	1	0	0
p.G119R	p.R406H	1	0	0
p.R474X	p.R406H	1	0	0
p.P50A	p.R406H	1	0	0
p.A240G	p.K441R	5	0	0
CFI VUS and VUS (*n* = 11)				
p.G261D	p.R406H	4	0	0
p.G261D	p.K441R	5	0	0
p.R406H	p.K441R	2	1[†]	0

[†]The p.R406H and p.K441R compound heterozygous participant with available OCT-derived macular measurements had a mean RPE thickness of 30.9 µm and a mean retinal thickness of 286.9 µm.
