# Supplementary material for: Rare complement factor I variants associated with reduced macular thickness and age-related macular degeneration in the UK Biobank
Source: Hum Mol Genet. 2022 Mar 14;31(16):2678–92. doi: 10.1093/hmg/ddac060 (PMC9402241; doi:10.1093/hmg/ddac060)
Supplement: Supplemental_Table_2_ddac060 [file supplemental_table_2_ddac060.pdf]

Supplemental Table 2. Slope analysis *P* values for association of RPE-BM and retinal macular thicknesses with age with genotype as an interactive term. Tukey's multiple comparison test was used for comparing *CFH* p.Y402H and *ARMS2* p.A69S subgroups. Individuals with a health record diagnosis of age-related macular degeneration or missing *CFI* genotype data were excluded. *P*<0.05 indicated with (*).

**Mean RPE-BM slope analysis, *P* value**				
**Genotype**	*CFI* type 1 RV	*CFI* VUS†	*CFH* p.Y402H	*ARMS2* p.A69S
**WT vs. Heterozygous**	0.38	0.68	0.26	0.03 *
**WT vs. Homozygous**	NA	NA	0.15	0.17
**Heterozygous vs. Homozygous**	NA	NA	0.73	0.81
**Mean retinal slope analysis, *P* value**				
**Genotype**	*CFI* type 1 RV	*CFI* VUS‡	*CFH* p.Y402H	*ARMS2* p.A69S
**WT vs. Heterozygous**	0.13	0.34	0.41	0.69
**WT vs. Homozygous**	NA	NA	0.22	0.35
**Heterozygous vs. Homozygous**	NA	NA	0.72	0.20

Abbreviations: RPE-BM = retinal pigment epithelium-Bruch’s membrane complex, RV = rare variant, WT = wild-type.

† Includes two *CFI* p.G261D homozygotes.

‡ Includes two *CFI* p.R406H homozygotes.
