# Supplementary material for: Rare complement factor I variants associated with reduced macular thickness and age-related macular degeneration in the UK Biobank
Source: Hum Mol Genet. 2022 Mar 14;31(16):2678–92. doi: 10.1093/hmg/ddac060 (PMC9402241; doi:10.1093/hmg/ddac060)
Supplement: Supplemental_Table_3_ddac060 [file supplemental_table_3_ddac060.pdf]

Supplemental Table 3. Odds ratios (ORs) of logistic regression analyses for AMD diagnosis, adjusted for age, gender, ethnicity, smoking status, and genotype and selected genotypes as fixed effects (multivariable OR), and site of recruitment as a random effect (multilevel OR).

**Dependent: AMD diagnosis**		**No AMD, N (%)**	**AMD, N (%)**	**Univariable OR (95% CI)**	**Multivariable OR (95% CI)**	**Multilevel OR (95% CI)**
***CFI* Type 1 RV**	Non-carrier	479,437 (99.2)	3,763 (0.8)	-	-	-
	Carrier	1,640 (98.3)	29 (1.7)	2.25 (1.52-3.19, *P*<0.001)	2.26 (1.50-3.25, *P*<0.001)	2.26 (1.56-3.27, *P*<0.001)
***CFI* VUS**	Non-carrier	475,169 (99.2)	3,742 (0.8)	-	-	-
	Carrier	6,725 (99.2)	52 (0.8)	0.98 (0.74-1.28, *P*=0.90)	1.00 (0.74-1.31, *P*=0.99)	1.00 (0.76-1.32, *P*=0.97)
***CFH* p.Y402H**	WT	186,453 (99.3)	1,277 (0.7)	-	-	-
	Heterozygous	227,000 (99.2)	1758 (0.8)	1.13 (1.05-1.22, *P*=0.001)	1.12 (1.04-1.21, *P*=0.002)	1.12 (1.04-1.21, *P*=0.002)
	Homozygous	69,925 (98.9)	780 (1.1)	1.63 (1.49-1.78, *P*<0.001)	1.63 (1.49-1.79, *P*<0.001)	1.64 (1.50-1.80, *P*<0.001)
***ARMS2* p.A69S**	WT	295,746 (99.3)	2,056 (0.7)	-	-	-
	Heterozygous	164,977 (99.2)	1407 (0.8)	1.23 (1.15-1.31, *P*<0.001)	1.23 (1.15-1.32, *P*<0.001)	1.23 (1.15-1.32, *P*<0.001)

	Homozygous	23,346 (98.5)	362 (1.5)	2.23 (1.99-2.49, *P*<0.001)	2.25 (2.01-2.53, *P*<0.001)	2.25 (2.01-2.53, *P*<0.001)
**Age (years)**	<50	117,736 (99.9)	137 (0.1)	-	-	-
	50 to 59	166,482 (99.6)	660 (0.4)	3.41 (2.84-4.11, *P*<0.001)	3.66 (3.02-4.46, *P*<0.001)	3.68 (3.05-4.45, *P*<0.001)
	≥60	214,336 (98.6)	3,153 (1.4)	12.64 (10.70-15.07, *P*<0.001)	13.66 (11.43-16.48, *P*<0.001)	13.73 (11.50-16.39, *P*<0.001)
**Gender**	Female	270,989 (99.1)	2,393 (0.9)	-	-	-
	Male	227,565 (99.3)	1557 (0.7)	0.77 (0.73-0.83, *P*<0.001)	0.74 (0.69-0.79, *P*<0.001)	0.74 (0.69-0.79, *P*<0.001)
**Ethnicity**	White	468,987 (99.2)	3,708 (0.8)	-	-	-
	Asian	9,787 (99.0)	95 (1.0)	1.23 (0.99-1.50, *P*=0.05)	1.82 (1.46-2.24, *P*<0.001)	2.05 (1.65-2.54, *P*<0.001)
	Black	7,999 (99.2)	62 (0.8)	0.98 (0.75-1.25, *P*=0.88)	1.65 (1.25-2.13, *P*<0.001)	1.80 (1.38-2.34, *P*<0.001)
	Chinese	1,563 (99.3)	11 (0.7)	0.89 (0.46-1.53, *P*=0.70)	1.44 (0.72-2.57, *P*=0.25)	1.53 (0.86-2.74, *P*=0.15)
	Mixed	2,944 (99.5)	14 (0.5)	0.60 (0.34-0.98, *P*=0.06)	0.81 (0.42-1.39, *P*=0.48)	0.85 (0.49-1.45, *P*=0.54)

	Other	4,527 (99.3)	31 (0.7)	0.87 (0.59-1.21, *P*=0.43)	1.24 (0.83-1.78, *P*=0.27)	1.33 (0.92-1.92, *P*=0.12)
**Smoking status**	Never	271,600 (99.3)	1,922 (0.7)	-	-	-
	Previous	171,407 (99.0)	1,649 (1.0)	1.36 (1.27-1.45, *P*<0.001)	1.18 (1.10-1.27, *P*<0.001)	1.18 (1.10-1.26, *P*<0.001)
	Current	52,627 (99.3)	351 (0.7)	0.94 (0.84-1.05, *P*=0.31)	1.11 (0.99-1.25, *P*=0.08)	1.12 (1.00-1.26, *P*=0.05)

Abbreviations: AMD = age-related macular degeneration, CI = confidence interval, OR = Odds ratio, RV = rare variant, VUS = variant of uncertain significance, WT = wild-type.
