# Supplementary material for: Rare complement factor I variants associated with reduced macular thickness and age-related macular degeneration in the UK Biobank
Source: Hum Mol Genet. 2022 Mar 14;31(16):2678–92. doi: 10.1093/hmg/ddac060 (PMC9402241; doi:10.1093/hmg/ddac060)
Supplement: Supplemental_Table_4_ddac060 [file supplemental_table_4_ddac060.pdf]

Supplemental Table 4. *CFI* rare variants present in this study.

Coding DNA sequence	Protein sequence (number with signal peptide)	Protein sequence (number without signal peptide)	Rs ID	Protein domain	Functional classification	Analyses with patient material	Analyses with recombinant protein	Other relevant studies
c.148C>G	p.P50A	p.P32A	rs144082872	FIMAC	Type 1	(1-4)	(1, 4, 5)	(6, 7)
c.355G>C	p.G119R	p.G101R	rs141853578	CD5	Type 1	(1-4, 8-11)	(4, 10)	(6, 12, 13)
c.485G>A	p.G162D	p.G144D	rs546607673	CD5	Type 1	(2, 3, 14-17)	(4)	
c.563G>T	p.G188V	p.G170V	rs769721080	CD5	Type 1 †	(4, 10)	(4, 10)	(18-20)
c.719C>G	p.A240G	p.A222G	rs146444258	LDLR1	Type 1	(2, 21, 22)	(4, 5)	(20, 23, 24)
c.859G>T	p.G287R	p.G269R	rs182078921	LDLR2	Type 1	(2)	(4)	(12, 13, 23)
c.1071T>G	p.I357M	p.I339M	rs200881135	SP domain	Type 1	(4, 18)	(4, 18)	(6, 17, 25-28)
c.1253A>T	p.H418L	p.H400L	rs121964912	SP domain	Type 1	(2, 18, 29)	(4, 18, 29)	(23, 30)
c.1291G>A	p.A431T	p.A413T	rs758017357	SP domain	Type 1	(1)	(1, 4)	(6, 24)
c.1420C>T	p.R474X	p.R456X	rs121964913	SP domain	Type 1	(1, 2, 4, 6, 8, 15)	(1, 4, 5)	(26, 31)
c.772+1G>T	-	-	rs775462739	-	Type 1‡	(2, 15, 18, 29, 32, 33)	N/A	(17, 28, 34)
c.1019T>C	p.I340T	p.I322T	rs769419740	SP domain	Type 2	(3, 14, 30, 35)	(4, 36)	(17, 20, 23, 24, 26-28)

c.782G>A	p.G261D	p.G243D	rs112534524	LDLR2	Type 3 / VUS	(1-4, 22, 37)	(4, 36, 37)	(1, 6, 12, 13, 20, 24, 28, 34)
c.1034G>A	p.R345Q	p.R327Q	rs746566803	SP domain	Type 3 / VUS	(38)	(4)	(13)
c.1217G>A	p.R406H	p.R388H	rs74817407	SP domain	Type 3 / VUS	(2, 4, 36, 39)	(4, 36, 39)	(13, 40)
c.1322A>G	p.K441R	p.K423R	rs41278047	SP domain	Type 3 / VUS	(2-4, 41)	(4)	(13, 20, 23, 24, 28, 42)

† Functional data on equivalent missense mutation, *CFI* c.563G>C (p.G188A), also considered for classification purposes.

‡ Null variant within the splice site of the gene. For classification purposes, we considered the studies referring to c.772G>A (r.658_773del) [rs199688124], a directly upstream splice site variant which leads to skipping of exon 5.

Abbreviations: FIMAC = factor I membrane attack complex, LDLR = low density lipoprotein receptor, SP = serine protease, VUS = variant of uncertain significance.

References

1 Bienaime, F., Dragon-Durey, M.-A., Regnier, C.H., Nilsson, S.C., Kwan, W.H., Blouin, J., Jablonski, M., Renault, N., Rameix-Welti, M.-A. and Loirat, C. (2010) Mutations in components of complement influence the outcome of Factor I-associated atypical hemolytic uremic syndrome. *Kidney Int.*, **77**, 339-349.

2 Kavanagh, D., Yu, Y., Schramm, E.C., Triebwasser, M., Wagner, E.K., Raychaudhuri, S., Daly, M.J., Atkinson, J.P. and Seddon, J.M. (2015) Rare genetic variants in the CFI gene are associated with advanced age-related macular degeneration and commonly result in reduced serum factor I levels. *Hum. Mol. Genet.*, **24**, 3861-3870.

3 Hallam, T.M., Marchbank, K.J., Harris, C.L., Osmond, C., Shuttleworth, V.G., Griffiths, H., Cree, A.J., Kavanagh, D. and Lotery, A.J. (2020) Rare Genetic Variants in Complement Factor I Lead to Low FI Plasma Levels Resulting in Increased Risk of Age-Related Macular Degeneration. *Invest. Ophthalmol. Vis. Sci.*, **61**, 18-18.

4 de Jong, S., Volokhina, E.B., de Breuk, A., Nilsson, S.C., de Jong, E.K., van der Kar, N.C.A.J., Bakker, B., Hoyng, C.B., van den Heuvel, L.P., Blom, A.M. *et al.* (2020) Effect of rare coding variants in the CFI gene on Factor I expression levels. *Hum. Mol. Genet.*, **29**, 2313-2324.

5 Nilsson, S.C., Kalchishkova, N., Trouw, L.A., Fremeaux-Bacchi, V., Villoutreix, B.O. and Blom, A.M. (2010) Mutations in complement factor I as found in atypical hemolytic uremic syndrome lead to either altered secretion or altered function of factor I. *Eur. J. Immunol.*, **40**, 172-185.

6 Fremeaux-Bacchi, V., Fakhouri, F., Garnier, A., Bienaimé, F., Dragon-Durey, M.-A., Ngo, S., Moulin, B., Servais, A., Provot, F. and Rostaing, L. (2013) Genetics and outcome of atypical hemolytic uremic syndrome: a nationwide French series comparing children and adults. *Clin. J. Am. Soc. Nephrol.*, **8**, 554-562.

7 Szilágyi, A., Kiss, N., Bereczki, C., Tálosi, G., Rácz, K., Túri, S., Györke, Z., Simon, E., Horváth, E., Kelen, K. *et al.* (2013) The role of complement in Streptococcus pneumoniae-associated haemolytic uraemic syndrome. *Nephrol. Dial. Transplant.*, **28**, 2237-2245.

8 Fakhouri, F., Roumenina, L., Provot, F., Sallée, M., Caillard, S., Couzi, L., Essig, M., Ribes, D., Dragon-Durey, M.A., Bridoux, F. *et al.* (2010) Pregnancy-associated hemolytic uremic syndrome revisited in the era of complement gene mutations. *J. Am. Soc. Nephrol.*, **21**, 859-867.

9 Servais, A., Noël, L.H., Roumenina, L.T., Le Quintrec, M., Ngo, S., Dragon-Durey, M.A., Macher, M.A., Zuber, J., Karras, A., Provot, F. *et al.* (2012) Acquired and genetic complement abnormalities play a critical role in dense deposit disease and other C3 glomerulopathies. *Kidney Int.*, **82**, 454-464.

10 van de Ven, J.P., Nilsson, S.C., Tan, P.L., Buitendijk, G.H., Ristau, T., Mohlin, F.C., Nabuurs, S.B., Schoenmaker-Koller, F.E., Smailhodzic, D., Campochiaro, P.A. *et al.* (2013) A functional variant in the CFI gene confers a high risk of age-related macular degeneration. *Nat. Genet.*, **45**, 813-817.

11 Geerlings, M.J., Kremlitzka, M., Bakker, B., Nilsson, S.C., Saksens, N.T., Lechanteur, Y.T., Pauper, M., Corominas, J., Fauser, S., Hoyng, C.B. *et al.* (2017) The Functional Effect of Rare Variants in Complement Genes on C3b Degradation in Patients With Age-Related Macular Degeneration. *JAMA Ophthalmol.*, **135**, 39-46.

12 Maga, T.K., Nishimura, C.J., Weaver, A.E., Frees, K.L. and Smith, R.J. (2010) Mutations in alternative pathway complement proteins in American patients with atypical hemolytic uremic syndrome. *Hum. Mutat.*, **31**, E1445-E1460.

13 Tan, P.L., Garrett, M.E., Willer, J.R., Campochiaro, P.A., Campochiaro, B., Zack, D.J., Ashley-Koch, A.E. and Katsanis, N. (2017) Systematic Functional Testing of Rare Variants: Contributions of CFI to Age-Related Macular Degeneration. *Invest. Ophthalmol. Vis. Sci.*, **58**, 1570-1576.

14 Le Quintrec, M., Lionet, A., Kamar, N., Karras, A., Barbier, S., Buchler, M., Fakhouri, F., Provost, F., Fridman, W. and Thervet, E. (2008) Complement mutation-associated de novo thrombotic microangiopathy following kidney transplantation. *Am. J. Transplant.*, **8**, 1694-1701.

15 Alba-Domínguez, M., López-Lera, A., Garrido, S., Nozal, P., González-Granado, I., Melero, J., Soler-Palacín, P., Cámara, C. and López-Trascasa, M. (2012) Complement factor I deficiency: a not so rare immune defect. Characterization of new mutations and the first large gene deletion. *Orphanet J. Rare. Dis.*, **7**, 42.

16 Franco-Jarava, C., Álvarez de la Campa, E., Solanich, X., Morandeira-Rego, F., Mas-Bosch, V., García-Prat, M., de la Cruz, X., Martín-Nalda, A., Soler-Palacín, P., Hernández-González, M. *et al.* (2017) Early Versus Late Diagnosis of Complement Factor I Deficiency: Clinical Consequences Illustrated in Two Families with Novel Homozygous CFI Mutations. *J. Clin. Immunol.*, **37**, 781-789.

17 El Sissy, C., Rosain, J., Vieira-Martins, P., Bordereau, P., Gruber, A., Devriese, M., de Pontual, L., Taha, M.-K., Fieschi, C., Picard, C. *et al.* (2019) Clinical and Genetic Spectrum of a Large Cohort With Total and Sub-total Complement Deficiencies. *Front. Immunol.*, **10**.

18 Nilsson, S.C., Trouw, L.A., Renault, N., Miteva, M.A., Genel, F., Zelazko, M., Marquart, H., Muller, K., Sjöholm, A.G. and Truedsson, L. (2009) Genetic, molecular and functional analyses of complement factor I deficiency. *Eur. J. Immunol.*, **39**, 310-323.

19 Alexander, P., Gibson, J., Cree, A.J., Ennis, S. and Lotery, A.J. (2014) Complement factor I and age-related macular degeneration. *Mol. Vis.*, **20**, 1253-1257.

20 Geerlings, M.J., Volokhina, E.B., de Jong, E.K., van de Kar, N., Pauper, M., Hoyng, C.B., van den Heuvel, L.P. and den Hollander, A.I. (2018) Genotype-phenotype correlations of low-frequency variants in the complement system in renal disease and age-related macular degeneration. *Clin. Genet.*, **94**, 330-338.

21 Caprioli, J., Noris, M., Brioschi, S., Pianetti, G., Castelletti, F., Bettinaglio, P., Mele, C., Bresin, E., Cassis, L. and Gamba, S. (2006) Genetics of HUS: the impact of MCP, CFH, and IF mutations on clinical presentation, response to treatment, and outcome. *Blood*, **108**, 1267-1279.

22 Servais, A., Frémeaux-Bacchi, V., Lequintrec, M., Salomon, R., Blouin, J., Knebelmann, B., Grünfeld, J.-P., Lesavre, P., Noël, L.-H. and Fakhouri, F. (2007) Primary glomerulonephritis with isolated C3 deposits: a new entity which shares common genetic risk factors with haemolytic uraemic syndrome. *J. Med. Genet.*, **44**, 193-199.

23 Seddon, J.M., Yu, Y., Miller, E.C., Reynolds, R., Tan, P.L., Gowrisankar, S., Goldstein, J.I., Triebwasser, M., Anderson, H.E., Zerbib, J. *et al.* (2013) Rare variants in CFI, C3 and C9 are associated with high risk of advanced age-related macular degeneration. *Nat. Genet.*, **45**, 1366-1370.

24 Bresin, E., Rurali, E., Caprioli, J., Sanchez-Corral, P., Fremeaux-Bacchi, V., Rodriguez de Cordoba, S., Pinto, S., Goodship, T.H.J., Alberti, M., Ribes, D. *et al.* (2013) Combined complement gene mutations in atypical hemolytic uremic syndrome influence clinical phenotype. *J. Am. Soc. Nephrol.*, **24**, 475-486.

25 Noris, M., Caprioli, J., Bresin, E., Mossali, C., Pianetti, G., Gamba, S., Daina, E., Fenili, C., Castelletti, F. and Sorosina, A. (2010) Relative role of genetic complement abnormalities in sporadic and familial aHUS and their impact on clinical phenotype. *Clin. J. Am. Soc. Nephrol.*, **5**, 1844-1859.

26 Westra, D., Volokhina, E., Van der Heijden, E., Vos, A., Huigen, M., Jansen, J., Van Kaauwen, E., Van der Velden, T., Van de Kar, N. and Van den Heuvel, L. (2010) Genetic disorders in complement (regulating) genes in patients with atypical haemolytic uraemic syndrome (aHUS). *Nephrol. Dial. Transplant.*, **25**, 2195-2202.

27 Geerdink, L.M., Westra, D., van Wijk, J.A.E., Dorresteijn, E.M., Lilien, M.R., Davin, J.-C., Kömhoff, M., Van Hoeck, K., van der Vlugt, A., van den Heuvel, L.P. *et al.* (2012) Atypical hemolytic uremic syndrome in children: complement mutations and clinical characteristics. *Pediatr. Nephrol.*, **27**, 1283-1291.

28 Fritsche, L.G., Igl, W., Bailey, J.N.C., Grassmann, F., Sengupta, S., Bragg-Gresham, J.L., Burdon, K.P., Hebbring, S.J., Wen, C., Gorski, M. *et al.* (2016) A large genome-wide association study of age-related macular degeneration highlights contributions of rare and common variants. *Nat. Genet.*, **48**, 134-143.

29 Vyse, T.J., Morley, B.J., Bartók, I., Theodoridis, E.L., Davies, K.A., Webster, A. and Walport, M.J. (1996) The molecular basis of hereditary complement factor I deficiency. *J. Clin. Invest.*, **97**, 925-933.

30 Shields, A.M., Pagnamenta, A.T., Pollard, A.J., , O., Taylor, J.C., Allroggen, H., Patel, S.Y., Taylor, J.C., Knight, S.J.L., Pagnamenta, A.T. *et al.* (2019) Classical and Non-classical Presentations of Complement Factor I Deficiency: Two Contrasting Cases Diagnosed via Genetic and Genomic Methods. *Front. Immunol.*, **10**.

31 Fremeaux-Bacchi, V., Dragon-Durey, M., Blouin, J., Vigneau, C., Kuypers, D., Boudailliez, B., Loirat, C., Rondeau, E. and Fridman, W. (2004) Complement factor I: a susceptibility gene for atypical haemolytic uraemic syndrome. *J. Med. Genet.*, **41**, e84.

32 Ponce-Castro, I.M., González-Rubio, C., Delgado-Cerviño, E.M., Abarrategui-Garrido, C., Fontán, G., Sánchez-Corral, P. and López-Trascasa, M. (2008) Molecular characterization of complement factor I deficiency in two Spanish families. *Mol. Immunol.*, **45**, 2764-2771.

33 Szarvas, N., Szilágyi, Á., Csuka, D., Takács, B., Rusai, K., Müller, T., Arbeiter, K., Réti, M., Haris, Á., Wagner, L. *et al.* (2016) Genetic analysis and functional characterization of novel mutations in a series of patients with atypical hemolytic uremic syndrome. *Mol. Immunol.*, **71**, 10-22.

34 Sullivan, M., Erlic, Z., Hoffmann, M.M., Arbeiter, K., Patzer, L., Budde, K., Hoppe, B., Zeier, M., Lhotta, K., Rybicki, L.A. *et al.* (2010) Epidemiological approach to identifying genetic predispositions for atypical hemolytic uremic syndrome. *Ann. Hum. Genet.*, **74**, 17-26.

35 Haerynck, F., Stordeur, P., Vandewalle, J., Van Coster, R., Bordon, V., De Baets, F., Schelstraete, P., Javaux, C., Bouvry, M.R., Fremeaux-Bacchi, V. *et al.* (2013) Complete factor I deficiency due to dysfunctional factor I with recurrent aseptic meningo-encephalitis. *J. Clin. Immunol.*, **33**, 1293-1301.

36 Kavanagh, D., Richards, A., Noris, M., Hauhart, R., Liszewski, M.K., Karpman, D., Goodship, J.A., Fremeaux-Bacchi, V., Remuzzi, G. and Goodship, T.H. (2008) Characterization of mutations in complement factor I (CFI) associated with hemolytic uremic syndrome. *Mol. Immunol.*, **45**, 95-105.

37 Nilsson, S.C., Karpman, D., Vaziri-Sani, F., Kristoffersson, A.C., Salomon, R., Provot, F., Fremeaux-Bacchi, V., Trouw, L.A. and Blom, A.M. (2007) A mutation in factor I that is associated with atypical hemolytic uremic syndrome does not affect the function of factor I in complement regulation. *Mol. Immunol.*, **44**, 1835-1844.

38 Fakhouri, F., Jablonski, M., Lepercq, J., Blouin, J., Benachi, A., Hourmant, M., Pirson, Y., Dürrbach, A., Grünfeld, J.-P., Knebelmann, B. *et al.* (2008) Factor H, membrane cofactor protein, and factor I mutations in patients with hemolysis, elevated liver enzymes, and low platelet count syndrome. *Blood*, **112**, 4542-4545.

39 Java, A., Pozzi, N., Love-Gregory, L.D., Heusel, J.W., Sung, Y.J., Hu, Z., Bertram, P., Liszewski, M.K., Cline, L.M., Ren, Z. *et al.* (2019) A Multimodality Approach to Assessing Factor I Genetic Variants in Atypical Hemolytic Uremic Syndrome. *Kidney Int. Rep.*, **4**, 1007-1017.

40 Yuasa, I., Nakagawa, M., Umetsu, K., Harihara, S., Matsusue, A., Nishimukai, H., Fukumori, Y., Saitou, N., Park, K.S., Jin, F. *et al.* (2008) Molecular basis of complement factor I (CFI) polymorphism: one of two polymorphic suballeles responsible for CFI A is Japanese-specific. *J. Hum. Genet.*, **53**, 1016-1021.

41 Caycı, F.S., Cakar, N., Hancer, V.S., Uncu, N., Acar, B. and Gur, G. (2012) Eculizumab therapy in a child with hemolytic uremic syndrome and CFI mutation. *Pediatr. Nephrol.*, **27**, 2327-2331.

42 Shoshany, N., Weiner, C., Safir, M., Einan-Lifshitz, A., Pokroy, R., Kol, A., Modai, S., Shomron, N. and Pras, E. (2019) Rare Genetic Variants in Jewish Patients Suffering from Age-Related Macular Degeneration. *Genes (Basel)*, **10**, 825.
