# Supplementary material for: Rare complement factor I variants associated with reduced macular thickness and age-related macular degeneration in the UK Biobank
Source: Hum Mol Genet. 2022 Mar 14;31(16):2678–92. doi: 10.1093/hmg/ddac060 (PMC9402241; doi:10.1093/hmg/ddac060)
Supplement: Supplemental_Table_5_ddac060 [file supplemental_table_5_ddac060.pdf]

Supplemental Table 5. Overview of logistic regression model assumptions for the prediction of age-related macular degeneration diagnosis. Age is included as a factor with terms <50, 50 to 59, and ≥60 years.

**Model**	**Included terms**									**Model fitting parameters**		
	**Fixed effects**								**Random effects**			
	***CFI* type 1 RV**	***CFI* VUS**	***CFH* Y402H**	***ARMS2* A69S**	**Age**	**Sex**	**Ethnicity**	**Smoking**	**Recruitment site**	**AIC**	**C-statistic**	**H&L test**
1	Yes	No	No	No	No	No	No	No	No	44333.5	0.502	0.00 (p=1.000)
2	Yes	Yes	No	No	No	No	No	No	No	44007.1	0.502	0.00 (p=1.000)
3	Yes	Yes	Yes	No	No	No	No	No	No	43790.1	0.542	0.12 (p=1.000)
4	Yes	Yes	Yes	Yes	No	No	No	No	No	43606.1	0.563	6.08 (p=0.638)
5	Yes	Yes	Yes	Yes	Yes	No	No	No	No	41281	0.725	7.01 (p=0.536)
6	Yes	Yes	Yes	Yes	Yes	Yes	No	No	No	41209.8	0.731	7.63 (p=0.470)
7	Yes	Yes	Yes	Yes	Yes	Yes	Yes	No	No	40886.6	0.733	6.47 (p=0.595)
8	Yes	Yes	Yes	Yes	Yes	Yes	Yes	Yes	No	40644.6	0.735	5.80 (p=0.669)
9	Yes	Yes	Yes	Yes	Yes	Yes	Yes	Yes	Yes	40458.8	0.747	N/A

Abbreviations: AIC = Akaike Information Criterion, H&L = Hosmer-Lemeshow, RV = rare variant.
