# Supplementary material for: Rare complement factor I variants associated with reduced macular thickness and age-related macular degeneration in the UK Biobank
Source: Hum Mol Genet. 2022 Mar 14;31(16):2678–92. doi: 10.1093/hmg/ddac060 (PMC9402241; doi:10.1093/hmg/ddac060)
Supplement: Supplemental_Table_6_ddac060 [file supplemental_table_6_ddac060.pdf]

Supplemental Table 6. Odds ratios (ORs) of multivariable logistic regression analyses for AMD diagnosis adjusted for age, gender, ethnicity, smoking status, and selected genotypes as fixed effects, fitted using maximum likelihood estimation, mean bias-reducing adjusted score, and median bias-reduction adjusted score equations (1).

**Dependent: AMD diagnosis**		**Maximum likelihood estimation, OR (95% CI)**	**Mean bias-reducing adjusted score, OR (95% CI)**	**Median bias-reducing adjusted score, OR (95% CI)**
***CFI* Type 1 RV**	Non-carrier	-	-	-
	Carrier	2.26 (1.50-3.25, *P*<0.001)	2.30 (1.57-3.37, *P*<0.001)	2.28 (1.55-3.34, *P*<0.001)
***CFI* VUS**	Non-carrier	-	-	-
	Carrier	1.00 (0.74-1.31, *P*=0.99)	1.01 (0.76-1.33, *P*=0.96)	1.00 (0.76-1.33, *P*=0.99)
***CFH* p.Y402H**	WT	-	-	-
	Heterozygous	1.12 (1.04-1.21, *P*=0.002)	1.12 (1.04-1.21, *P*=0.002)	1.12 (1.04-1.21, *P*=0.002)
	Homozygous	1.63 (1.49-1.79, *P*<0.001)	1.63 (1.49-1.79, *P*<0.001)	1.63 (1.49-1.79, *P*<0.001)
***ARMS2* p.A69S**	WT	-	-	-
	Heterozygous	1.23 (1.15-1.32, *P*<0.001)	1.23 (1.15-1.32, *P*<0.001)	1.23 (1.15-1.32, *P*<0.001)
	Homozygous	2.25 (2.01-2.53, *P*<0.001)	2.26 (2.01-2.53, *P*<0.001)	2.26 (2.01-2.53, *P*<0.001)
**Age (years)**	<50	-	-	-
	50 to 59	3.66 (3.02-4.46, *P*<0.001)	3.64 (3.00-4.43, *P*<0.001)	3.65 (3.01-4.44, *P*<0.001)
	≥60	13.7 (11.4-16.5, *P*<0.001)	13.6 (11.4-16.4, *P*<0.001)	13.6 (11.4-16.4, *P*<0.001)
**Gender**	Female	-	-	-
	Male	0.74 (0.69-0.79, *P*<0.001)	0.74 (0.69-0.79, *P*<0.001)	0.74 (0.69-0.79, *P*<0.001)
**Ethnicity**	White	-	-	-
	Asian	1.82 (1.46-2.24, *P*<0.001)	1.83 (1.47-2.26, *P*<0.001)	1.82 (1.47-2.25, *P*<0.001)
	Black	1.65 (1.25-2.13, *P*<0.001)	1.66 (1.27-2.17, *P*<0.001)	1.65 (1.27-2.16, *P*<0.001)
	Chinese	1.44 (0.72-2.57, *P*=0.25)	1.51 (0.82-2.80, *P*=0.19)	1.47 (0.79-2.74, *P*=0.23)
	Mixed	0.81 (0.42-1.39, *P*=0.48)	0.84 (0.47-1.51, *P*=0.56)	0.82 (0.45-1.47, *P*=0.50)
	Other	1.24 (0.83-1.78, *P*=0.27)	1.26 (0.87-1.85, *P*=0.23)	1.25 (0.85-1.83, *P*=0.25)
**Smoking status**	Never	-	-	-
	Previous	1.18 (1.10-1.27, *P*<0.001)	1.18 (1.10-1.27, *P*<0.001)	1.18 (1.10-1.27, *P*<0.001)
	Current	1.11 (0.99-1.25, *P*=0.08)	1.11 (0.99-1.25, *P*=0.08)	1.11 (0.99-1.25, *P*=0.08)

Abbreviations: AMD = age-related macular degeneration, CI = confidence interval, OR = Odds ratio, RV = rare variant, VUS = variant of uncertain significance, WT = wild-type.

References

1 Kosmidis, I., Kenne Pagui, E.C. and Sartori, N. (2020) Mean and median bias reduction in generalized linear models. *Stat. Comput.*, **30**, 43-59.
